# Dysregulation of TNF-α and IFN-γ expression is a common host immune response in a chronically infected mouse model of melioidosis when comparing multiple human strains of *Burkholderia pseudomallei*

**DOI:** 10.1186/s12865-020-0333-9

**Published:** 2020-02-03

**Authors:** Kei Amemiya, Jennifer L. Dankmeyer, Jeremy J. Bearss, Xiankun Zeng, Spencer W. Stonier, Carl Soffler, Christopher K. Cote, Susan L. Welkos, David P. Fetterer, Taylor B. Chance, Sylvia R. Trevino, Patricia L. Worsham, David M. Waag

**Affiliations:** 1 Bacteriology Division, US Army Medical Research Institute of Infectious Diseases, Fort Detrick, Frederick, MD USA; 2 Pathology Division, US Army Medical Research Institute of Infectious Diseases, Fort Detrick, Frederick, MD USA; 3 Virology Division, US Army Medical Research Institute of Infectious Diseases, Fort Detrick, Frederick, MD USA

**Keywords:** *Burkholderia pseudomallei*, Chronic infection, BALB/c mice, Pyogranulomatous lesion, Inflammatory cytokines, Intracellular TNFα and IFNγ

## Abstract

**Background:**

Melioidosis is endemic in Southeast Asia and Northern Australia and is caused by the Gram-negative, facultative intracellular pathogen *Burkholderia pseudomallei*. Diagnosis of melioidosis is often difficult because of the protean clinical presentation of the disease, and it may mimic other diseases, such as tuberculosis. There are many different strains of *B. pseudomallei* that have been isolated from patients with melioidosis, but it was not clear if they could cause a similar disease in a chronic BALB/c murine model of melioidosis. Hence, we wanted to examine chronically infected mice exposed to different strains of *B. pseudomallei* to determine if there were differences in the host immune response to the pathogen.

**Results:**

We identified common host immune responses exhibited in chronically infected BALB/c mice, although there was some heterogeneity in the host response in chronically infected mice after exposure to different strains of *B. pseudomallei*. They all displayed pyogranulomatous lesions in their spleens with a large influx of monocytes/macrophages, NK cells, and neutrophils identified by flow cytometry. Sera from chronically infected mice by ELISA exhibited elevated IgG titers to the pathogen, and we detected by Luminex micro-bead array technology a significant increase in the expression of inflammatory cytokines/chemokines, such as IFN-γ, IL-1α, IL-1β, KC, and MIG. By immunohistochemical and in situ RNA hybridization analysis we found that the increased expression of proinflammatory cytokines (IL-1α, IL-1β, TNF-α, IFN-γ) was confined primarily to the area with the pathogen within pyogranulomatous lesions. We also found that cultured splenocytes from chronically infected mice could express IFN-γ, TNF-α, and MIP-1α ex vivo without the need for additional exogenous stimulation. In addition by flow cytometry, we detected significant amounts of intracellular expression of TNF-α and IFN-γ without a protein transport blocker in monocytes/macrophages, NK cells, and neutrophils but not in CD4^+^ or CD8^+^ T cells in splenocytes from chronically infected mice.

**Conclusion:**

Taken together the common features we have identified in chronically infected mice when 10 different human clinical strains of *B. pseudomallei* were examined could serve as biomarkers when evaluating potential therapeutic agents in mice for the treatment of chronic melioidosis in humans.

## Background

Melioidosis is not a well-known disease that is endemic in Southeast Asia and Northern Australia, although it is the third leading cause of disease in that area after tuberculosis and HIV/AIDS. It is caused by the Gram-negative, facultative intracellular, bacterium *Burkholderia pseudomallei*, which may be found in soil and wet lands. Infected patients most often present with symptoms of pneumonia and may have multiple abscesses [1, 2]. There are multiple risk factors that appear to be associated with melioidosis, and the most common has been identified as diabetes mellitus which is followed by excess alcohol consumption, renal disease, and lung disease [3, 4]. Although these risk factors signal the possible presence of immunosuppressive conditions in the patient, HIV/AIDS patients do not appear to be more susceptible to infection by *B. pseudomallei* [5, 6]. There also appears to be a sex (male) and age (> 45 years) bias to the disease in the endemic area. Presently, there is a growing epidemic of diabetes in the general population that could potentially lead to an increase in the incidence of melioidosis. Because melioidosis has not been considered or recognized until recently in the clinic, a patient with melioidosis that exhibits common symptoms that are associated with other diseases, such as tuberculosis, may be misdiagnosed [7, 8]. Treatment of melioidosis is intensive and prolonged because of the intrinsic resistance mechanisms of *B. pseudomallei* to certain classes of antibiotics and because of the ability of the pathogen to establish a latent or chronic infection [9–13]. Outside of Northern Australia, there is a high mortality in patients diagnosed with melioidosis [1, 2]. Currently, there is no efficacious human vaccine available against *B. pseudomallei* infection. Because of the ease of growth and dissemination of the pathogen, and the clinical outcome after exposure to the pathogen, *B. pseudomallei* has been considered a Category B select agent by the Center of Disease Control in the United States.

Pneumonia is the most common clinical presentation of melioidosis, which suggests that inhalation of *B. pseudomallei* may be one of the common routes of infection [1, 2]. Other potential routes of infection are through percutaneous infection from exposure to the presence of *B. pseudomallei* in contaminated water or soil or ingestion of contaminated food or water. The initial contact of the host with *B. pseudomallei* by aerosol exposure may be through the large to small airways of the lungs and into the alveolar compartment where resident macrophages (and dendritic cells) reside to phagocytize the pathogen [14, 15]. *B. pseudomallei* has been shown to infect both phagocytic and nonphagocytic cells, so it could also infect bronchial and pulmonary epithelial cells in the airways [16–20]. Activation of the host’s innate immune system by the pathogen in epithelial and resident macrophages (or phagocytes) can occur through recognition by the host of the pathogen associated molecular patterns (PAMPS) presented by *B. pseudomallei*. These could potentially trigger the host’s Toll-like receptors (TLR) 2, 4, and 5 that are pattern-recognition receptors that recognize PAMPs consisting of peptidoglycan, lipopolysaccharide, and flagellin molecules, respectively [2, 18]. Through MyD88-dependent and possibly MyD88-independent signal transduction pathways, the induction and expression of inflammatory cytokines expression, such as IFN-γ, IL-1α, IL-1β, IL-6, IL-18, and TNF-α may be induced [21–24]. Subsequent spread of *B. pseudomallei* from the lungs may occur hematogenously to the lymph nodes, spleen, and liver, either as free organisms and/or within phagocytic cells, such as macrophages (or dendritic cells) or neutrophils.

There has been an increase in reported cases of melioidosis because of a recent heightened awareness of the disease [25, 26]. However, there appears to be a large variety of strains of *B. pseudomallei* that has been attributed to cause melioidosis in humans which may be partly responsible for the diverse clinical picture presented by the disease. In addition, genetic heterogeneity in specific strains of *B. pseudomallei* could be detected even in a single patient in an acute or chronic case of melioidosis [27, 28]. This underscored the importance of getting a better understanding of different clinical strains of *B. pseudomallei* with their interaction with the host [25]. In a recent study a number of human clinical isolates of *B. pseudomallei* with documented low passage number were collected, and their virulence was evaluated in a 21 day median lethal dose (LD_50_) study in a BALB/c murine model of melioidosis after aerosol exposure [29]. This study presented us with the unique opportunity to examine chronically infected BALB/c mice after the 21 day LD_50_ aerosol study to ascertain if there were differences in the murine host response when many different clinical strains of *B. pseudomallei* were evaluated under similar conditions. In previous murine studies of melioidosis, usually one or two strains of *B. pseudomallei* were evaluated at the same time [30, 31]. Furthermore, we have shown that the BALB/c mouse is very susceptible to infection to all the human clinical strains of *B. pseudomallei* examined, while the C57BL/6 mouse was significantly more resistant to infection to the same *B. pseudomallei* strains compared to the BALB/c mouse [29]. In the present report, we present the results of the BALB/c host immune response in chronically infected survivors after aerosol exposure to 10 separate human clinical strains of *B. pseudomallei*. We examined both humoral and cell-mediated immune responses to the pathogen in aerosol exposed, chronically infected mice. We were able to identify a number of host immune responses or characteristics that were common in the chronically infected BALB/c mice exposed to at least 10 different human clinical strains of *B. pseudomallei* when compared with naive mice in a mouse model of chronic melioidosis.

## Results

### Examination of chronically infected BALB/c mice after aerosol exposure to different strains of *B. pseudomallei*

In a previous study, BALB/c mice were exposed to different clinical isolates of *B. pseudomallei* (Table 1) to determine their 21 day aerosol LD_50_ to assess their relative virulence [29]. In the present study we examined survivors when they became available after the 21 day aerosol exposure study and examined their sera and spleens to evaluate their immune response to the infection (see Additional file 1: Figure S1). We examined 4–7 surviving mice after exposure to each strain from the same colony forming unit (CFU) exposure group whenever possible. Although there were differences in the time post-infection (PI) that we examined survivors after they were initially exposed (22–70 days PI), we still found apparently uninfected and chronically infected mice in many cases from the same exposure group. The number of survivors in each exposure group was influenced by the virulence of the *B. pseudomallei* strain that was evaluated [29]. We focused our studies on sera and spleens from the exposed mice because of the large number of samples that had to be processed and examined, and the spleens were a good representative organ of the course of infection because they became infected soon (1–2 days) after exposure to the pathogen [29, 30]. We validated the mice that were chronically infected by isolation of the pathogen from their spleens after the initial 21 day LD_50_ study.

**Table 1:** Human clinical strains of *B. pseudomallei* (Bp) evaluated in BALB/c mice

	Bp Strain^a^	Country of Origin	Source/Clinical History
1	MSHR5855	Australia	Pneumonia, sputum sample
2	HBPUB10134a	Thailand	Septicemia/pneumonia
3	496e	Thailand	Toe-swab, male, disseminated
4	1026b	Thailand	Blood, diabetic female, disseminated
5	MSHR668	Australia	Blood, male, encephalomyelitis
6	MSHR5848	Australia	Pneumonia, sputum sample
7	MSHR5858	Australia	Septicemia/pneumonia, sputum sample
8	Bp22^b^	Singapore	Male, pneumonia/sepsis
9	MSHR305	Australia	Encephalomyelitis
10	K96243	Thailand	Blood, diabetic female, septicemia
11	1106a	Thailand	Disseminated, female, liver abscess

^a^ Trevino et al., 2018 [29]

^b^ Formally strain named KHW

**Fig. 1:**
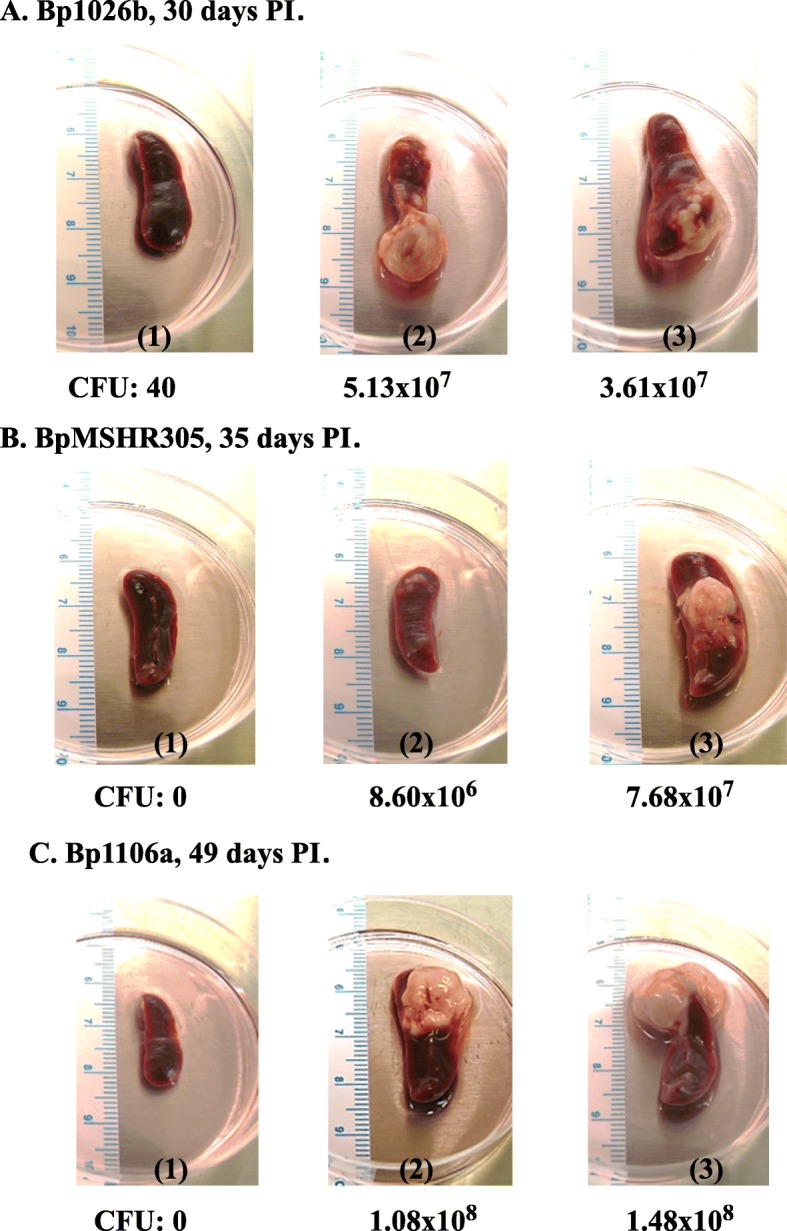
Example of spleens from survivors after aerosol exposure to clinical strains of *B. pseudomallei* shows heterogeneity of infection. Spleens recovered from surviving mice after exposure to the follow Bp strains, the amount of exposure, and the number of days PI: **A**, Bp1026b, exposed to 2 CFU, 30 days PI; **B**, BpMSHR305, exposed to 3 CFU, 35 days PI; and **C**, Bp1106a, exposed to 2851, 49 days PI. Below each spleen examined was the number of CFU recovered

Figure 1 shows examples of some spleens examined from survivors from the aerosol exposure studies showing heterogeneity in infection. We examined survivors after they were exposed to 2 CFU of *B. pseudomallei* 1026b, 30 days PI (Fig. 1a). We recovered 40, 5.13 × 10^7^, and 3.61 × 10^7^ CFU from spleens shown in panels 1, 2, and 3, respectively. In Fig. 1b we examined survivors after exposure to 3 CFU of *B. pseudomallei* MSHR305, 35 days PI. In this case we recovered 0, 8.60 × 10^6^, and 7.68 × 10^7^ CFU from spleens shown in panels 1, 2, and 3, respectively. In Fig. 1c, we examined survivors after exposure to 2851 CFU of *B. pseudomallei* 1106a, 49 days PI. We recovered 0, 1.08 × 10^8^, and 1.48 × 10^8^ CFU from spleens in panels 1, 2, and 3, respectively. In summary, we recovered spleens from aerosol exposed mice that were heterogeneous in appearance that could be placed into 3 general categories based on size, CFU recovered, and the appearance of a pyogranulomatous lesion. These categories were: Gp 2, normal appearing spleens, 0 CFU recovered; Gp 3, enlarged/swollen spleens without a visable pyogranulomatous lesion, and 0 to few CFU recovered; and Gp 4, enlarged spleens with a visable pyogranulomatous lesion(s), and a large number of CFU recovered (> 1000) (chronically infected). Spleens from naïve mice that served as unexposed controls for comparison were placed into Gp 1.

Table 2 is a summary of the different categories of spleens recovered and analyzed from BALB/c mice exposed to each of the 11 individual clinical strains of *B. pseudomallei* with their LD_50_ to show relative virulence and how they relate to the other strains [29]. Generally, with mice that were exposed to the most virulent *B. pseudomallei* strains MSHR5855 and HBPUB10134a, with reported LD_50_ of 0.35 and 0.99 CFU, respectively, we saw more surviving mice with normal appearing spleens than mice with chronically infected spleens (presence of pyogranulomatous lesion(s) and isolated CFU) because most mice in the latter group that were acutely infected did not survive past 21 days PI. On the other hand, with mice exposed to the least virulent strain (*B. pseudomallei* 1106a (LD_50_ 4266 CFU) we saw more chronically infected mice. We also listed the number of mice that survived in the group(s) of mice from which we obtained mice that we evaluated. We analyzed the immune state of each individual mouse after exposure to the different strains of *B. pseudomallei* (see below). (Although we were not able to examine mice after aerosol exposure to *B. pseudomallei* HBPUB10134a because they died by 21 days PI,) We had previously reported on the acute infection of BALB/c mice by *B. pseudomallei* HB10134a [29, 32].

**Table 2:** Types of spleens isolated from BALB/c mice after aerosol exposure to clinical isolates of *B. pseudomallei* (Bp)

				No. Spleens Recovered in each Category^a,b^		
	Bp Strain	LD_50_ (CFU)^c^	No. survivors left in exposure group (s) considered ^d^	Normal appearing Gp 2	Enlarged, Swollen Gp 3	Chronic, Infected, pyogranulomatous lesion Gp 4
1	BpMSHR5855	0.35	9/20	7	0	1
2	BpHBPUB10134a	0.99	11/20	8	0	0^e^
3	Bp406e	2.79	6/20	3	1	2
4	Bp1026b	4.10	8/10	0	1	3
5	BpMSHR668	4.42	7/20	5	1	1
6	BpMSHR5848	4.66	13/20	6	1	1
7	BpMSHR5858	4.97	5/10	0	0	4
8	Bp22^f^	5.00	11/20	5	0	2
9	BpMSHR305	5.98	6/10	0	1	3
10	BpK96243	25.1	13/20	3	1	2
11	Bp1106a	4266	10/20	0	2	8^g^
	Total:			37	8	27

^a^ Naïve, unexposed BALB/c mice (*n* = 10) are in Group 1

^b^ Spleens were recovered 22–70 days post-infection

^c^ LD_50_ from [29]

^d^ Number of survivors from one exposure group (initially 10 mice) or two exposure groups (initially 20 mice) from where we obtained mice to evaluate after 21 days

^e^ No infected survivors were recovered because they died before day 21

^f^ LD_50_ was determined late and not reported in [29]

^g^ This group includes 2 infected spleens that were used for immunohistochemical analysis

### Elevated antibody response in chronically infected BALB/c mice

We wanted to examine the antigen specific antibody level in survivors from the 21 day aerosol study after exposure to the different clinical strains of *B. pseudomallei* to evaluate the host humoral immune response to the pathogen. We examined the levels of *B. pseudomallei* specific IgG and the subclasses IgG1 and IgG2a in sera of BALB/c mice by ELISA that survived aerosol exposure to different strains of *B. pseudomallei*. Table 3 shows the antibody response in sera from mice arranged into groups according to the characteristics of the spleens that we recovered after the 21 day aerosol study. Results of antibody titers from sera that were repeated but not acceptable, for example if the reading was inhibited, or not complete were not included.

**Table 3:** Chronically infected mice show elevated antibody levels after infection with clinical strains of *B. pseudomallei*

Group^b^		Antibody Titer^a^		Ratio
	IgG	IgG1	IgG2a	IgG2a/IgG1
Gp. 1. Naïve (10)	50 (1.00)^c^	50 (1.00)^c^	50 (1.00)^c^	1.00
Gp. 2. Normal Appearing (32)^e^	58 (1.10)	53 (1.05)	50 (1.00)^c^	0.94
Gp. 3. Swollen (8)^e^	28,537 (2.45) *P* = 0.0002^d^	10,089 (2.12) *P* = 0.0002^d^	22,307 (3.17) *P* = 0.0019^d^	2.21
Gp. 4. Chronically Infected(23)^e^	201,364 (1.24) *P* < 0.0001^d^	68,046 (1.33) *P* < 0.0001^d^	82,328 (1.34) *P* < 0.0001^d^	1.21

^a^ Antibody titers were done in triplicate for each mouse at least once, and titers are reported as geometric mean with geometric SEM

^b^ There was a total of 63 sera from exposed BALB/c mice. The number in parentheses represents the number of mice in each group

^c^ The number 50 was used for calculation of statistics although the antibody titers were less than 50

^d^ The significant levels are derived from comparing antibody levels for specific groups with those of naïve (Gp 1) mice

^e^ The exposure strain and number of mice (in parenthesis) in each group as follows: Gp. 2, MSHR5855 (7), HBPUB10134a (8), 406e (3), MSHR668 (5), MSHR5848 (6), K96243 (3); Gp. 3, 406e (1), 1026b (1), MSHR668 (1), MSHR5848 (1), MSHR305 (1), K96243 (1), 1106a (2); Gp. 4, MSHR5855 (1), 406e (2), 1026b (3), MSHR668 (1), MSHR5848 (1), MSHR5858 (4), MSHR305 (3), K96243 (2), 1106a (6)

We saw little difference in the antibody IgG titer of BALB/c mice with normal appearing spleens (Gp 2) compared to that of naïve mice in Gp.1. In mice that we recovered enlarged spleens (Gp3) we saw a significant increase in the antibody IgG titer (*P* = 0.0002) and the subclasses IgG1 (*P* = 0.0002) and IgG2a titers (*P* = 0.0019) compared to naïve mice. There was a further increase in the IgG titer when we examined the sera from mice with chronically infected spleens (Gp 4). When we compared the ratio of the subclass IgG2a/IgG1 responses for Gp 3 and Gp 4, we found that mice with spleens in Gp 3 had a modest Th1-like response, while the ratio for the subclass response for mice with spleens in Gp 2 and Gp 4 were more of an equal Th1- to Th2-like response.

### Increased cellular host response in spleens from chronically infected mice

**Fig. 2:**
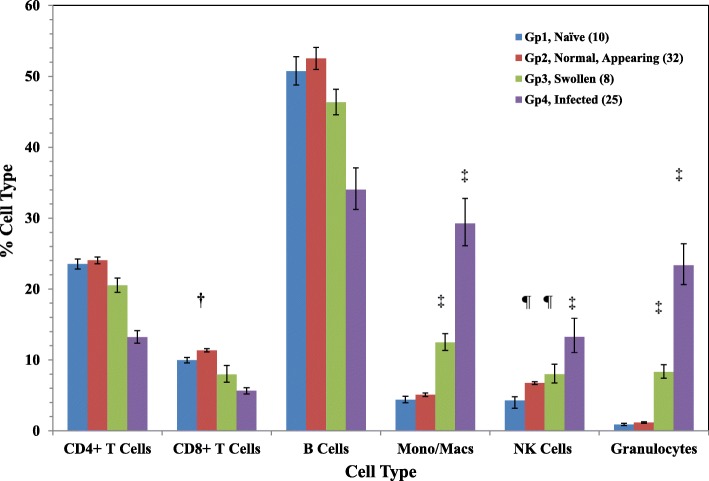
Cellular composition of BALB/c spleens recovered after aerosol exposure to clinical strains of *B. pseudomallei*. Spleen cells were prepared as described in Material and Methods and labeled for CD4+ and CD8+ T cells, B cells, monocyte/macrophages, NK cells, and granulocytes for identified by flow cytometry. The data was from the analysis of a total of 75 spleens from 4 groups of spleens. A comparision of the composition of isolated spleens placed into different groups is shown: Gp 1, naïve; Gp 2, normal appearing; Gp 3, enlarged swollen; and Gp 4, chronically infected. The results are reported as the geometric mean of the percentage of cells with the standard error of the mean. The number in parenthesis after each group represents the number of spleens in each group, and each determination was completed at least once for each spleen. For spleens from naïve mice (*n* = 10), the cellular composition was the following: CD4^+^ T cells, 23.51% (1.03); CD8^+^ T cells, 9.96% (1.04); B cells, 50.7% (1.04); monocytes/macrophages, 4.38% (1.11); NK cells, 4.29% (1.12); and granulocytes, 0.89% (1.19)%. The significant levels compared to the cells from naïve mice (Gp 1) as shown: †, *P* < 0.05; ¶, *P* < 0.01; ‡, *P* ≤ 0.0001. The type and number of each strain (in parenthesis) in each group are as follows: Gp. 2, MSHR668 (5), 406e (3), K96243 (3), MSHR5855 (7), MSHR (5848 (6), HBPUB10134a (8); Gp. 3, 406e (1), 1106a (2), MSHR305 (1), MSHR668 (1), K96243 (1), 1026b (1), MSHR5854 (1); Gp. 4, MSHR668 (1), 406e (2), 1106a (6), MSHR305 (3), K96243 (2), 1026b (3), Bp22 (2), MSHR5858 (4), MSHR5855 (1), MSHR5848 (1)

To evaluate the cellular host response in BALB/c mice after exposure to different strains of *B. pseudomallei*, we used flow cytometry to examine the cellular composition of spleens from chronically infected survivors. We assessed the amount of CD4^+^ and CD8^+^ T cells, B cells, monocytes/macrophages, NK cells, and granulocytes in spleens of surviving BALB/c mice and compared them to the cellular composition of spleens from naïve mice (Gp 1) (Fig. 2). Results of flow cytometry readings that were repeated when possible but not acceptable, such as readings were inhibited, or not complete were not included. There was little change in the cellular composition of spleens from BALB/c mice placed into Gp 2 (normal appearing, *n* = 32) compared to spleens from naïve mice. However, we saw small but notable increases in the percentages of CD8^+^ T cells and NK cells compared to the amounts in naïve spleens (*P* = 0.0114 and *P* = 0.0028, respectively). In enlarged spleens from BALB/c mice survivors belonging to Group 3 (*n* = 8) we observed a modest increase in the relative amounts of monocyte/macrophages (*P* < 0.0001), NK cells (*P* = 0.0085), and granulocytes (*P* < 0.0001) compared to spleens from naïve mice. In infected spleens (Gp 4) with visible pyogranulomatous lesions (*n* = 25), we found a substantial increase (*P* < 0.0001) in the percentage of monocyte/macrophages [29.26% (1.12)], NK cells [13.26% (1.20)], and granulocytes [23.25% (1.13)].

### Increased host inflammatory immune response in chronically infected mice

**Fig. 3:**
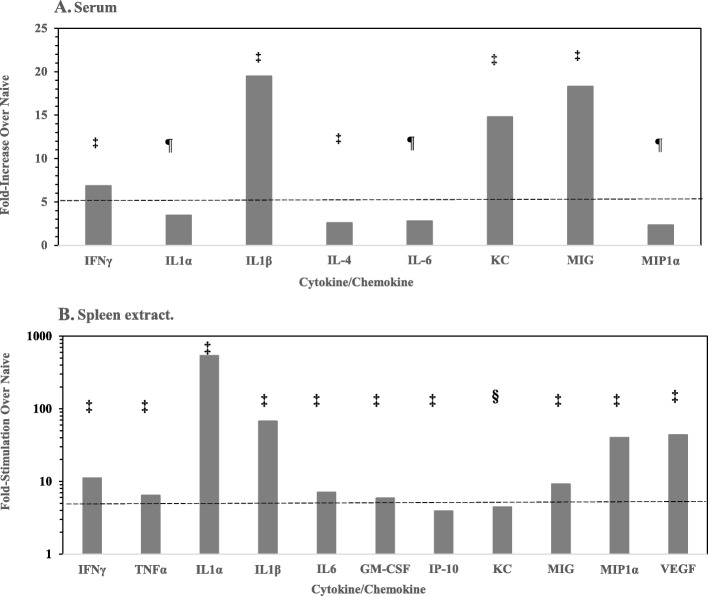
Cytokine/chemokine analysis of sera and spleen preparations from BALB/c mice after aerosol exposure to clinical strains of *B. pseudomallei*. The cytokine/chemokine levels in a total of 63 surviving mice were evaluated at least once in duplicate in addition to 10 naïve mice. Levels of up to twenty of cytokines/chemokines were evaluated although the results of only 17/20 are shown for the different groups (Additional file 2: Table S1). **A.** Only 9/17 cytokines/chemokines were significantly elevated in sera from mice with chronically infected spleens (Gp 4) compared to that in sera from naïve mice (Gp 1), and those that were elevated more than 2-fold over naïve mice are shown. **B.** In spleen extracts the amounts of 14/17 cytokines/chemokines were significantly elevated in spleens from chronically infected mice (Gp 4), and we show 11/14 that were more than 2-fold over the levels in spleen extracts from naïve mice. Note the difference in the scale in the fold-increase between the sera and spleen extracts. The dotted line represents 5-fold increase in amounts in both figures for perspective. The significant differences are reported between the amount in naïve mice versus the amount in chronically infected mice (Gp 4): ¶, *P* < 0.01; §, *P* < 0.001; ‡, *P* < 0.0001

We wanted to examine the host inflammatory immune response in chronically infected survivors after exposure to different strains of *B. pseudomallei*. We thus examined the presence of inflammatory cytokines/chemokines in sera and spleen extracts from the aerosol exposed survivors after the 21 day aerosol study by Luminex technology (Additional file 2: Table S1). Results of Luminex readings that were repeated when possible but not acceptable, such as readings were not within the standard, or not complete were not included. We assessed 20 cytokines/chemokines in the sera of selected survivors, and we observed no significant increases in the level of cytokines/chemokines in sera from mice with normal appearing spleens (Gp 2, *n* = 32) compared to that in sera from naïve mice (Gp 1, *n* = 10). Of the cytokines/chemokines we examined in sera of BALB/c mice with enlarged spleens (Gp. 3, *n* = 8) none were elevated over that found in sera from naive mice (Gp. 1). In contrast, some cytokines/chemokines were significantly elevated (9/17 shown) in sera from mice with chronically infected spleens (Gp. 4, *n* = 23) compared to amounts in serum from naive mice (Gp. 1). These were IFNγ, IL-1α, IL-1β, IL-4, IL-6, IL-12, KC, MIG, and MIP-1α. Those cytokines/chemokines found in sera that were increased greater than 2-fold over that in naïve mice are shown in Fig. 3A (8/20 examined).

In contrast to sera, we saw a substantial increase in the number and amount of cytokines/chemokines present in spleen extracts from BALB/c mice that survived the 21 day aerosol study (see Additional file 2: Table S1). In spleen extracts prepared from normal appearing spleens in Gp 2 (*n* = 32), 3/17 cytokines/chemokines were modestly increased over that in spleen extracts from naïve mice in Gp. 1 (IL-1α, IL-1β, and MIG). In spleen extracts prepared from enlarged spleens in Gp. 3 (*n* = 8) there was a notable increase in 5/17 of the cytokines/chemokines (IL-1α, IL-1β, MIG, MIP-1a, and VEGF). In contrast, in spleen extracts prepared from chronically infected mice in Gp.4 (*n* = 23), the levels of 14/17 cytokines/chemokines were markedly increased (Additional file 2: Table S1). We showed in Fig. 3B the cytokines/chemokines (11) that were greater than 2-fold elevated over that in spleen extracts from naïve mice. They were GM-CSF, IFN-γ, TNF-α, IL-1α, IL-1β, IL-6, IP-10, KC, MIG, MIP-1α, and VEGF. Those that were not more than 2-fold elevated over that in naïve mice although were significantly increased were IL-2, IL-5, and MCP-1. The major elevated inflammatory cytokines/chemokines in both sera and spleen extracts were IFN-γ IL-1α, IL-1β, IL-6, KC, MIG, and MIP-1α (see Additional file 2: Table S1; and Fig. 3A, B).

When we examined the correlation of Th2-like cytokines (IL-4, IL-5, IL-10) to the Th1-like IFNγ in sera of Gp 3 mice, there was a modest Th1-like response (1.81-fold, the average of the fold-increase of the 3 cytokines divided into the fold-increase of IFNγ), which was similar to the IgG2a/IgG1 ratio (2.21) (see Additional file 2: Table S1 and Table 3, respectively, although we did not see this response in extracts from mice in Gp 3. When we examined the same relationship of cytokines in the sera of Gp 4 mice, we saw an overall higher Th1-like response (4.6-fold), which was more positive than the Th1/Th2 ratio of the antibody subclass response (1.21). At the same time when the relationship of these cytokines to the subclass response was examined in the spleen extracts from chronically infected Gp 4 mice, the Th1-like response was even overall higher (10.9-fold). Thus overall the cellular immune response in mice chronically infected with *B. pseudomallei* was noticeably toward a Th1-like response compared to what we observed with the antibody response.

### Enhanced expression of proinflammatory cytokines is closely associated with the pathogen in pyogranulomatous lesions

**Fig. 4:**
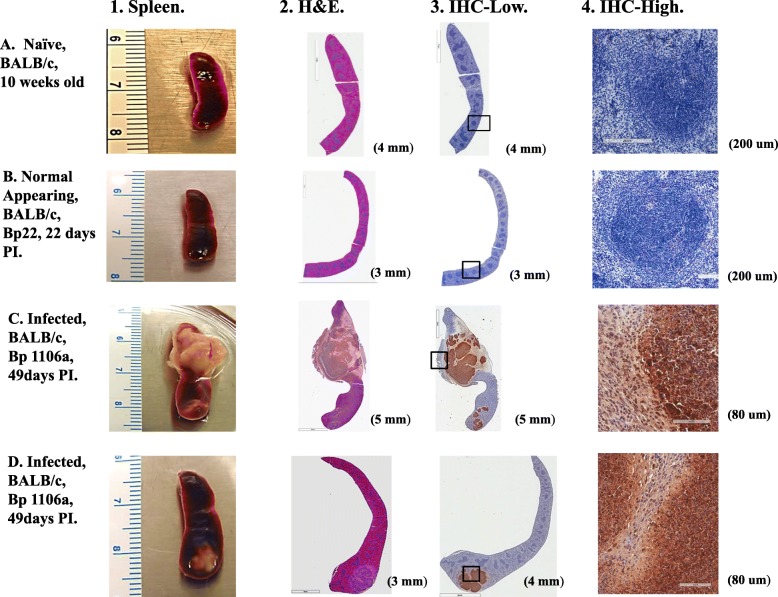
Histochemical and immunohistochemical analysis of spleens from mice exposed by aerosol to *B. pseudomallei* shows the pyogranulomatous lesions and the presence of the pathogen. Figures are representative of 16 separate spleens from 6 studies containing 60 mice (5 naïve mice and 11 exposed mice). Results are shown for the following spleens: **A**. a spleen from a naïve BALB/c mouse, 10 weeks old; **B**. a normal appearing spleen from a BALB/c mouse exposed to 1 CFU of B*. pseudomallei* 22 after 22 days PI; **C** and **D** infected spleens from BALB/c mice exposed to 2851 CFU of *B. pseudomallei* 1106a after 49 days PI, one grossly infected and the other an early minor infected spleen, respectively. Column 1 shows the picture of the respective spleens before analysis. Column 2 shows paraffin sections of the respective spleens stained with hematoxylin and eosin (H&E) that shows the presence of pyogranulomatous lesions present in the infected spleens (B,C,D). Scale bar shown on lower right. Column 3 shows the presence and location of immunoreactive exopolysaccharide material of *B. pseudomallei* present in infected spleens at low magnification (IHC-Low) relative to the whole spleen. Scale bar shown on lower right. Column 4 shows a higher magnification (IHC-High) of the immunoreactive material on the edge of pyogranulomatous lesions with stained epithelial macrophages surrounding some lesions. Scale bar shown on lower right

We saw in *B. pseudomallei* chronically infected mice (Gp. 4) that we could recover high CFU from spleens, and we also could detect the expression of significant amounts of the proinflammatory cytokines IL-1α, IL-1β, TNF-α, and IFN-γ in their spleens. We wanted to investigate the cellular location of the pathogen relative to the location where the proinflammatory cytokines were expressed in the chronically infected spleens. To reveal the presence of *B. pseudomallei* in situ in these spleens, we probed the spleens with a high titer rabbit antibody that reacted with the exopolysaccharide of *B. pseudomallei* [33–35]. A total of 15 sections of different spleens were stained with hematoxylin and eosin (H&E), and 16 spleen sections were probed with an anti-*B. pseudomallei* rabbit antibody (naïve, normal appearing, and chronically infected spleens from *B. pseudomallei* strains 22, K96243, or 1106a). In Fig. 4 we show representative spleens from BALB/c mice that are (A) a spleen from a naïve BALB/c mouse, (B) a normal appearing spleen (from *B. pseudomallei* Bp22 exposed), and (C and D) two chronically infected spleens (from *B. pseudomallei* 1106a exposed). In the normal appearing spleen (B) we did not see any exopolysaccharide positive staining material (Fig. 4B, panels 3 and 4) by immunohistochemical (IHC) analysis. These are similar to results that we saw when spleens from naïve mice were examined (Fig. 4A). In the examples of infected spleens from chronically infected BALB/c mice (Fig. 4C and D) we saw immunoreactive staining exopolysaccharide material associated with pyogranulomatous lesions that were nodular and largely multifocal that were primarily composed of a mixture of neutrophils, macrophages, and lymphocytes often with a thin surrounding layer of epithelioid macrophages (Fig. 4C and D, panels 3 and 4). We choose to show the spleen in Fig. 4D because it represented a good example of an early pyogranuloma lesion, compared with the spleen in Fig. 4C which showed a gross pyogranuloma, even though the mice were both exposed to Bp 1106a. We also observed the pathogen in multifocal pyogranulomatous lesions encased within a single large capsule-like structure that may displace in some cases more than half the spleen, in addition to separate individual pyogranulomatous lesions (see Fig. 4C).

**Fig. 5:**
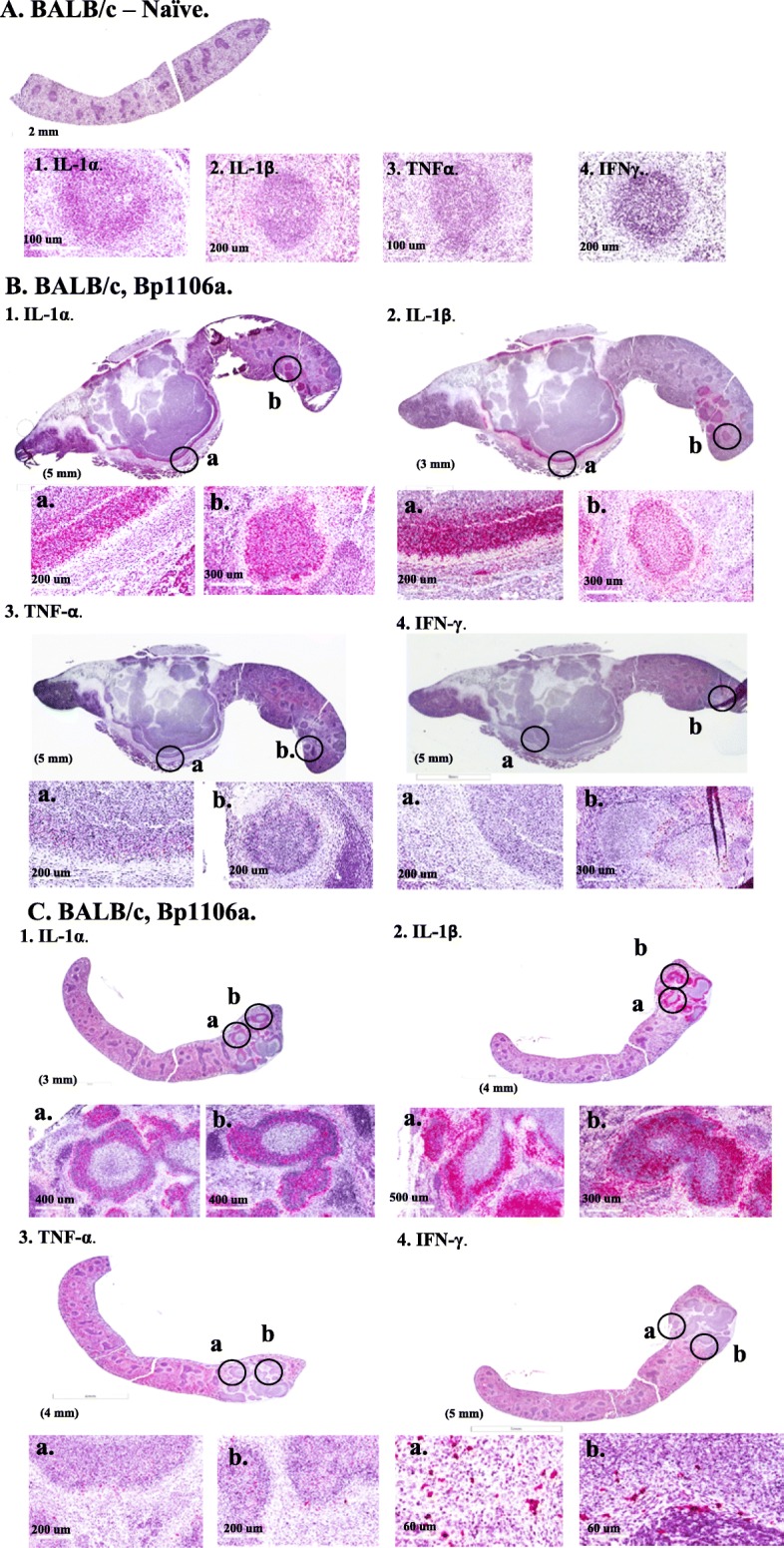
Detection of inflammatory cytokine expression in *B. pseudomallei* exposed mouse spleens. A total of 12 spleens were each examined at least once with 4 different RNA hybridization probes: 2 spleens from naïve BALB/c mice, 5 normal appearing spleens from exposed BALB/c, and 5 infected spleens from BALB mice. Examples of results from a control naïve mouse and 2 infected spleens from 2 BALB/c are shown. In situ RNA hybridization was used to detect the expression of IL-1α (panel 1), IL-1β (panel 2), TNF-α (panel 3), and IFN-γ (panel 4) in the spleens. **A**. A spleen from a control naïve mouse is shown in **A**. **B**. A spleen from a BALB/c mouse exposed to 2851 CFU of *B. pseudomallei* 1106a 49 days PI with a massive, multifocal pyogranulomatous growth occupying more than half of the spleen. Many reactive cells can be seen for IL-1α, IL-1β, TNF-α RNA probes at the outer edge of a capsular structure containing multiple, unreactive pyogranulomatous nodules and in separate pyogranulomatous lesions. Much fewer scattered IFN-γ reactive cells could be seen in the same areas. **C**. A spleen from a BALB/c mouse exposed to 2851 CFU of *B. pseudomallei* 1106a 49 days PI, showing an early development of a multifocal pyogranulomatous lesion. Many reactive cells can be seen for IL-1α, IL-1β, and TNF-α in the outer edge of pyogranulomatous lesions. Many reactive cells can also be seen in the fibrotic epithelial macrophage region surrounding the pyogranulomatous lesions. Few IFN-γ reactive cells can be seen near the pyogranulomatous lesion

We then wanted to determine the location of the proinflammatory cytokines relative to the location of the pathogen in spleens from exposed mice. In the following study and those below, we showed by different methods that we could detect the expression of proinflammatory cytokines, specifically TNF-α and IFN-γ expression in splenocytes from chronically infected mice. Because of the difficulty in obtaining reliable antibodies to probe protein targets in formalin-fixed, paraffin-embedded tissue, we used an in situ RNA hybridization method to locate the expression of proinflammatory cytokines (IL-1α, IL-1β, TNF-α, IFN-γ) in spleens from *B. pseudomallei* exposed mice [36]. We examined a total of 12 spleens with each set of 4 different cytokine RNA probes: 2 naïve spleens; 5 normal appearing spleens (exposed to *B. pseudomallei* Bp22 or K96243); or 5 infected spleens (exposed to *B. pseudomallei* K96243 or 1106a). In Fig. 5B and C we show examples of in situ RNA hybridization to detect the expression of IL-1α, IL-1β, TNF-α, and IFN-γ in two *B. pseudomallei* 1106a infected BALB/c spleens, 49 days PI. In Fig. 5A, panels 1–4, we show the results of the in situ RNA hybridization with the 4 RNA probes with a spleen from a naïve mouse where no concentrated cluster or pattern of a positive signal was seen with the probes. In Fig. 5B and C, panels 1–3, we saw at least two types of staining with the RNA probes for IL-1α, IL-1β, and TNF-α. First, there was a positive signal that appeared to be predominately located within the outer edge of an encapsulated structure that contained multifocal pyogranulomatous lesions. The multifocal pyogranulomatous lesions within the large encapsulated structure did not stain with any of cytokine RNA probes tested although they were positive for the presence of the exopolysaccharide (see Fig. 4C). Some epithelioid macrophages within the fibrotic region surrounding the pyogranulomatous encapsulated region also stained with the IL-1α, IL-1β, and TNF-α RNA probes. The second type of staining we saw with the three cytokine probes were individual pyogranulomatous lesions located in different areas of the spleen. The three RNA probes also hybridized to most of the lesion with some in cells in the immediate surrounding area (Fig. 5B, C, panels 1–3). With the IFN-γ RNA probe, however, we saw fewer cells stained than with the other three cytokine probes in the infected spleen (Fig. 5B and C, panels 4), and the few IFN-γ stained cells also appeared to be located near the pyogranulomatous lesions. No other region further outside of the pyogranulomatous lesions showed many positive cells arranged in a similar pattern as the proinflammatory cytokine RNA probes. Overall, we saw intense abundant staining with the IL-1α and IL-1β RNA probes within the pyogranulomatous lesion or within the capsule structure surrounding pyogranulomatous lesions. We observed less intense but still abundant staining with the TNF-α RNA probe in the same areas. We saw much fewer cells stained with the IFN-γ RNA probe that were not arranged in the same pattern as the other cytokine probes. In comparison, in spleens from naïve mice we saw few to moderate number of cells scattered throughout the spleen that were positive with the IL-1α, IL-1β, and TNF-α RNA probes but not any with the IFN-γ RNA probe (Fig. 5A). There was no organized pattern to these signals as seen in spleens with pyogranulomatous lesions. In summary, the presence of both the pathogen and the heightened expression of proinflammatory cytokines were both generally confined within the pyogranulomatous lesion in infected spleens from chronically infected mice.

### Ex vivo expression of IFN-γ, TNF-α, and MIP-1α by splenocytes from chronically infected mice

Since we recovered live microorganisms from spleens from chronically infected BALB/c mice after aerosol exposure to different strains of *B. pseudomallei*, it suggested to us that spleen cells in the infected spleens were persistently stimulated by the presence of the pathogen, and therefore, they would be actively expressing cytokines/chemokines in response to the pathogen, and we should be able to detect this activity ex vivo. We began these series of studies after we started examining the chronically infected mice, and realized that there was a possibility that splenocytes could be immunologically activated. We were able to examine 8 infected spleens (see below) to support our hypothesis. To investigate this theory, we cultured spleen cells from exposed mice ex vivo under three culture conditions without the presence of a protein transport blocker: 1) with media only (unstimulated), 2) with inactivated *B. pseudomallei* whole-cells, or 3) with phorbol myristate acetate (PMA) and ionomycin as a positive control. After 2 days of incubation, we measured the presence of cytokines/chemokines in the supernatant. In Table 4 we demonstrated the activation of splenocytes by presenting the results of spleen cells (total of 20 preparations shown, that includes 2 from naïve mice) prepared from BALB/c mice exposed to 9 different strains of *B. pseudomallei* (1106a, 406e, MSHR668, 1026b, MSHR5858, MSHR5855, MSHR5848, HBPUB10134a, and Bp22) and arranged into the three different groups according to the characteristic of the spleen (see Fig. 2). We also showed the results of spleen cells from two naïve mice (Gp 1) incubated under the same conditions for comparison. With spleen cells from naïve mice we saw very little difference in the amount of IFN-γ expressed when incubated with the whole-cell antigen or without the antigen (media only), while with PMA and ionomycin we saw a substantial amount of IFN-γ produced. With spleen cells from normal appearing spleens (Gp. 2, *n* = 7) in the media only culture condition little IFN-γ was produced, and when these spleen cells where incubated with the whole-cell antigen there was a small increase of IFN-γ produced but not significant when compared to cells from naïve mice. However, a similar amount of IFN-γ was produced by these spleen cells when incubated with PMA and ionomycin as seen with spleen cells from naïve mice. Spleen cells prepared from enlarged spleens (Gp.3, *n* = 3) produced little to moderate amounts of IFN-γ in media only conditions, but they produced significant amounts of IFN-γ (*P* < 0.0100), after restimulated with whole-cell antigen [5297 (1.27) pg/ml] compared to naïve cells or slightly higher amounts of IFN-γ with PMA and ionomycin [6381 (1.26) pg/ml]. In contrast, we saw significant amounts of IFN-γ produced (*P* < 0.0010) by spleen cells prepared from infected spleens (Gp. 4, *n* = 8) in the presence of media only [4440 (1.48) pg/ml] compared to spleen cells from naïve mice. IFN-γ was also produced by these spleen cells when they were restimulated with the whole-cell antigen [2727 (1.27) pg/ml, *P* < 0.0009)] and slightly higher amounts with PMA [6117 (1.17) pg/ml. At the same time, we also measured the expression of two other cytokines as examples that no exogenous antigen was needed for their expression by splenocytes from chronically infected mice. In Table 4 we showed the amount of TNF-α and MIP-1α we found in the same cultures as we examined for IFN-γ. However, with these two inflammatory cytokine/chemokines we did not see consistent expression or stimulation in the same cultural conditions as we did for IFN-γ.

**Table 4:** Ex vivo expression of IFN-γ, TNF-α, and MIP-1α by stimulated and unstimulated spleen cells

Group/ State of Spleen^b,e^	IFN-γ Expressed (pg/ml)^a^			TNF-α Expressed (pg/ml)			MIP-1α Expressed (pg/ml)		
	Media Only	Antigen^c,d^	PMA+Iono	Media Only	Antigen	PMA+Iono	Media Only	Antigen	PMA+Iono
Gp. 1. Naïve (2)	16.0 (1.00)	26.7 (1.40)	5373 (1.05)	8.00 (1.02)	115.9 (1.07)	546.0 (1.04)	31.6 (1.24)	177.8 (1.11)	9,502 (1.21)
Gp. 2. Normal Appearing (7)	17.2 (1.08)	136.0 (2.17)	4850 (1.09)	12.1 (1.29)	194.1 (1.21)	622.6 (1.74)	29.2 (1.15)	772.2 (1.54)	11,261 (1.79)
Gp. 3. Swollen (3)	142.1 (3.05)	5297 (1.27)^¶^	6381 (1.26)	56.6 (2.23)	790.9 (1.11)^§^	163.2 (2.29)	144.5 (3.54)	5,235 (1.83)^†^	734.7 (4.58)
Gp. 4. Chronic, Infected (8)	4440 (1.48)^§^	2727 (1.55)^¶^	6117 (1.17)	2998 (1.56)^§^	2543 (1.22)^‡^	6752 (1.44)^†^	11270 (1.60)^§^	5289 (1.66)^†^	33264 (1.25)^†^

^a^Cytokine/chemokine determinations were all done at least once in duplicate and reported as geometric means with geometric SEMs

^b^A total of 18 spleens from aerosol exposed BALB/c mice were placed into different categories, and the number in parentheses represents the number of samples in each group

^c^The stimulation antigen was the exposure strain used in the aerosol studies that was inactivated

^d^The significant levels shown represent differences between results from naïve mice (Gp1) and results of the other groups under the same conditions: ^†^*P* < 0.05; ^¶^*P* < 0.01; ^§^*P* < 0.001; ^‡^*P* < 0.0001

^e^The exposure strain and number of mice (in parenthesis) in each group are as follows: Gp. 2, 406e (1), MSHR668 (1), MSHR5855 (1), MSHR5848 (1), HBPUB10134a (2), Bp22 (1); Gp. 3, 1106a (1), MSHR668 (1), 1026b (1); Gp. 4, 1106a (1), 406e (1), 1026b (1), MSHR5858 (2), MSHR5855 (1), MSHR5848 (1), Bp22 (1)

### Detection of intracellular expression of TNF-α and IFN-γ in inflammatory cells in spleens from chronically infected mice

To further explore the idea that spleen cells from *B*. *pseudomalle*i chronically infected BALB/c mice were persistently stimulated, we wanted to examine spleen cells from chronically infected mice for the intracellular expression of TNF-α and IFN-γ, and at the same time identify the type of cells that were expressing the cytokines by flow cytometry. These two cytokines play an important role in protection and pathogenesis in melioidosis [37–41]. Similar to the previous ex vivo study where these studies were started after we examined chronically infected mice, we were able to demonstrate this phenomenon in 14 chronically infected spleens that were infected by 7/10 different strains of *B. pseudomallei* (see below), as well as 28 normal appearing spleens and 7 swollen spleens. Results from spleens that were not complete were not included. For this study spleen cells were neither exogenously stimulated before examination nor were inhibitors of intracellular transport/secretion, such as Brefeldin A or Monensin used [42–44]. Examples of the flow cytometric analysis of intracellular TNF-α or IFN-γ in spleen cells from different types of spleens from aerosol exposed BALB/c mice are shown in Additional file 3: Figure S2AB, respectively.

**Fig. 6:**
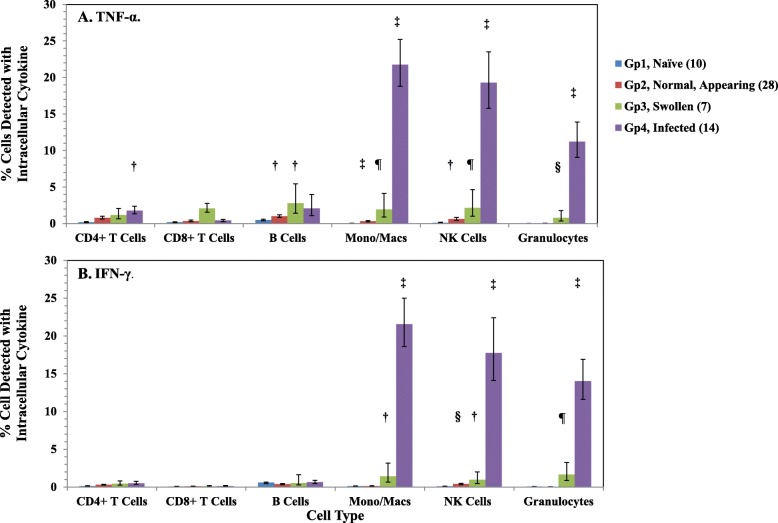
Intracellular expression of TNF-α and IFN-γ in spleen cells from *B. pseudomallei* exposed BALB/c mice. Examples of spleen cells (grouped according to their characteristics, see Fig. 1) from a total of at least 49 *B. pseudomallei* (included 8 different strains) aerosol exposed BALB/c mice and from 10 naïve BALB/c mice were analyzed for the intracellular expression of TNF-α (**A**) and IFN-γ (**B**) are shown. Intracellular cytokine expression was detected with anti-IFN-γ-APC or anti-TNF-γ-APC after cells were surface labeled and permeabilized. The results are reported as the percentage of cell type (geometric means with SEM) containing TNF-α or IFN-γ, and analysis of each spleen was determined at least once. The percentage of cell type (geometric mean with SEM) in naïve BALB/c mice that expressed intracellular TNF-α/IFN-γ were as follows: CD4+ T cells, 0.17 (1.45)%/0.13 (.132)%; CD8+ T cells, 0.17 (1.41)%/0.06 (1.30)%; B cells, 0.48 (1.30)%/0.59 (1.11)%; monocyte/macrophages, 0.05 (1.25)%/0.12 (1.23)%; NK cells, 0.09 (1.87)%/0.09 (1.39)%; granulocytes, 0.01 (1.74)%/0.06 (1.18)%. The type and number of each strain (in parenthesis) in each group are as follows: Gp. 2, MSHR668 (5), 406e (3), K96243 (3), MSHR5855 (7), MSHR5848 (6), HBPUB10134a (4); Gp. 3, 406e (1), 1106a (2), MSHR305 (1), MSHR668 (1), 1026b (1), MSHR5854 (1); Gp. 4, MSHR668 (1), 406e (1), 1106a (6), K96243 (1), 1026b (3), MSHR5855 (1), MSHR5848 (1). The significant levels compared to that in naïve cells are shown: †, *P* < 0.05; ¶, *P* < 0.01; §, *P* < 0.001; ‡, *P* ≤ 0.0001

Figure 6A shows the results of the cytometric analysis of the presence of intracellular TNF-α in spleen cells from 49 BALB/c mice that were exposed to different strains of *B. pseudomallei*, and compared them with the analysis of spleen cells from naïve mice (*n* = 10). Spleen cells from naïve BALB/c mice (Gp 1) showed very low levels of intracellular TNF-α in all the cell types that were examined (CD4^+^ T cells, CD8^+^ T cells, B cells, monocyte/macrophages, NK cells, and granulocytes). In spleen cells from normal appearing spleens (Gp. 2, *n* = 28, exposed to *B. pseudomallei* strains 406e, K96243, MSHR668, MSHR5855, MSHR5848, and HBPUB10134a), we detected small increases of intracellular TNF-α in CD4^+^ and CD8^+^ T cells and detected a modest increase in the percentage of B cells (*P* = 0.0349) with intracellular TNF-α compared to that in spleen cells from naïve mice. We saw a further increase in the percentages of monocyte/macrophages (*P* < 0.0001), and NK cells (*P* = 0.0186) that contained intracellular TNF-α, but not in granulocytes from normal appearing spleens. In spleen cells from enlarged spleens (Gp. 3, *n* = 7, exposed to *B. pseudomallei* strains 406e, 1106a, MSHR305, MSHR668, 1026b, and MSHR5854, we saw small increases in the percentage of CD4^+^ and CD8^+^ T cells with intracellular TNF-α, and a modest increase in the number of B cells with intracellular TNF-α. However, we detected small but significant increases in the number of monocyte/macrophages (*P* = 0.0021), NK cells (*P* = 0.0080), and granulocytes (*P* = 0.0007) that contained TNF-α. In infected spleens (Gp. 4, *n* = 14, exposed to *B. pseudomallei* strains 406e, 1026b, 1106a, K96243, MSHR668, MSHR5848, and MSHR5855) we observed a notable increase in the percentage of CD4^+^ T cells (*P* = 0.0417) but not in CD8^+^ T cells or in B cells that contained TNF-α. In contrast, we detected a significant (*P < 0.0001*) number of monocyte/macrophages [21.8 (1.16)%], NK cells [19.3 (1.22)%], and granulocytes [11.23 (1.24)%] that contained TNF-α compared to spleen cells from naïve mice.

When we examined the same spleen cells for the presence of intracellular IFN-γ (Fig. 6B), we saw very little IFN-γ expressed in all cell types from naïve BALB/c mice (Gp. 1). In spleen cells from normal appearing spleens (Gp. 2) there was very little IFN-γ detected, except in NK cells (*P* = 0.0008) compared to that present in naïve spleen cells. We saw an increase in the percentage of cells that contained intracellular IFN-γ in enlarged spleens (Gp. 3), specifically in monocyte/macrophages (*P* = 0.0189), NK cells (*P* = 0.0160), and granulocytes (*P* = 0.0020), but not in CD4^+^ or CD8^+^ T cells or in B cells. When we examined cells from infected spleens (Gp. 4), we saw a significant increase (*P <* 0.0001) in the percentage of monocyte/macrophages [21.6 (1.16)%], NK cells [17.8 (1.26)%], and granulocytes [14.0 (1.21)%] that contained intracellular IFN-γ compared to that in naïve spleen cells, but not in CD4^+^ and CD8^+^ T cells or in B cells. We have summarized the findings in the present study when comparing different strains of *B. pseudomallei* in the chronically infected BALB/c mouse model of melioidosis (Table 5).

**Table 5:** Characteristics of spleens/sera in BALB/c mice after aerosol exposure to strains of *B. pseudomallei*

General Properties		Category of Spleens from Aerosol Exposed Mice		
	Naïve	Normal appearing	Enlarged	Chronically Infected
	Gp 1^a^	Gp 2	Gp 3	Gp 4
Weight, size	~ 100 mg	~ 100 mg	> 100 mg - 400+ mg	> 400 mg - > 1500 mg
Pyogranulomatous (visable) lesion	No	No	No	Yes
CFU recovered	No	No	0–240	10^3^ - 10^9^
Elevated IgG response	No	No, or very low	Yes	Yes
Increased inflammatory cells	No	Very little^b^	Yes^c^	Yes^c^
Increased cytokine/ chemokine expression				
Serum	No	No	No	Yes
Spleen extract	No	Yes^d^	Yes	Yes
Ex vivo expression cytokines	No	Very little^e^	Yes	Yes ^f^
Intracellular cytokine expression				
TNF-α	No	Very little	Yes	Yes
IFN-γ	No	No	Yes	Yes

^a^ Served as control for all characteristics/properties of other groups

^b^ Includes CD8+ T cells, monocytes/macrophages, and NK cells

^c^ Includes monocytes/macrophages, NK cells, and neutrophils

^d^ Consist of IL-1α, IL-1β, and MIG

^e^ Not statistically different

^f^ Already stimulated

## Discussion

Our ability to detect the intracellular expression of TNF-α and IFN-γ in inflammatory cells from infected spleens in the present study was independent of the presence of an intracellular protein transport inhibitor, such as Brefeldin A (BFA) [45], and it was a common phenomenon observed in mice that were chronically infected with different human clinical strains of *B. pseudomallei*. Detection of intracellular expression of IFN-γ has been previously reported in CD8^+^ T cells in a viral (although limited amounts) infection [46] or in CD4+ and CD8+ T cells in *Mycobacterium tuberculosis* infection in the lung of mice without BFA treatment [47]*.* Because *B. pseudomallei* is a facultative intracellular pathogen, the over expression of TNF-α and IFN-γ may be the direct consequence of the host response to the pathogen. There may be a number of events or processes that may have contributed to our observation. One event could be the persistent presence of the intracellular pathogen could be stressful to the host cell [2, 16, 19] that could lead to the disruption of the normal processing of proteins within the infected cell. Host secretory proteins which are folded and post-translationally modified within the endoplasmic reticulum (ER) may be affected by the presence of an intracellular pathogen, and proteins may become incorrectly folded and may accumulate in the ER to induce a condition termed “ER stress”. Another pathway that may be affected by the presence of the intracellular pathogen is the transport of proteins between the ER and Golgi, which is the pathway blocked by BFA [45]. Thus, our ability to detect the intracellular expression of IFN-γ and TNF-α in cells from chronically infected mice may be the consequence of one or more events that may induce cell stress, or possibly from the interference of the normal processing of host proteins or acquisition of proteins within the host cell by the presence and /or activity of *B. pseudomallei*. The expression of IFN-γ and TNF-α may be considered important for the early protective innate immune response by the host to *B. pseudomallei*, but in chronic stages of infection in melioidosis, the expression of IFN-γ and TNF-α (as well as IL-1α and IL-1β) appears to be part of the immunopathology which occurs at this later stage of infection [48–54].

We also noted that in the chronically infected spleens, little or no intracellular expression of IFN-γ or TNF-α was seen in CD4^+^ or CD8^+^ T cells compared to the amount of these two cytokines seen in monocytes/macrophages, NK cells, or granulocytes. This observation was in contrast to the reports of cell-mediated immune responses in clinical cases of melioidosis that correlated with survival or a positive outcome in acute cases of the disease [40, 55, 56]. This difference could be that our results were derived from a chronic infection model of melioidosis (in BALB/c mice). Under these conditions, our inability to detect the intracellular expression of IFN-γ or very little amounts of TNF-α in CD4+ and CD8+ T cells may be in part the consequence of T cell exhaustion that has been observed before in chronic viral and bacterial infections, and in tumors [57, 58]. In addition, program cell death-1 (PD-1), which is a checkpoint inhibitory receptor, was found on the dysfunctional T cell population that increased in number at a later stage of infection. In a chronic (6 months) murine model for brucellosis it was reported that CD8+ T cells became nonfunctional or exhausted and could not express IFN-γ after stimulation with a purified MHC class I *Brucella* specific peptide when compared to CD8+ T cells prepared from spleens from acutely (2 weeks) infected mice [59]. In addition, the exhausted CD8+ T cell population also expressed inhibitor receptors, such as PD-1 and lymphocyte activation gene 3. In an in vitro study on the effect of *B. pseudomallei* K96243 on human polymorphonuclear neutrophils (PMNs) and CD4+ T cell function, it was found that there was a greater inhibition of T cell functions (proliferation and IFN-γ expression) by infected *B. pseudomallei* PMNs than by uninfected PMNs [60]. It was also reported that T cell suppression caused by infected PMNs was mediated by the programmed death 1 (PD-1)/programmed death ligand-1 (PD-L1) pathway. There was also a correlation in the increase of PD-L1 on PMNs with decreased T cell activity in patients with Type 2 diabetes. In a murine model of melioidosis, it was found that infection of mice with a small colony variant (SCV) of *B. pseudomallei* resulted in a higher bacterial load 60 days PI than a normal size colony wild-type strain [61]. Furthermore, PD-1 expression but not cytotoxic T lymphocyte-associated protein 4 (CTLA-4) was upregulated on CD4+ and C8+ T cells in PBMCs in mice persistently infected with the SCV of *B. pseudomallei,* in addition to a simultaneous decrease in the number of CD4+ T cells. Further studies are needed to support our finding that in *B. pseudomallei* chronically infected mice that T-cells function may be inhibited in part from the consequence of the persistent intracellular presence of *B. pseudomallei* in host cells, which may lead to the possible expression of PD-1 or CTLA-4 on T-cells in chronic melioidosis. The presence of these checkpoint inhibitors on T-cells might be another common feature that is associated with chronic melioidosis.

There was a previous report on a chronic murine model for melioidosis reported by Conejero et al. (2011) [35]. Although they reported a similar development of splenomegaly and pyogranuloma formation after infection as we observed, however, there were several major differences from our present study. One was the use of C57BL/6 mice compared to BALB/c mice in the present study. Another was the route of infection was by intranasal inoculation rather than whole-body aerosol exposure used in our study. And a third difference was Conejero et al. (2011) used *B. pseudomallei* 576, an isolate from a human fatal case of melioidosis in Thailand, which we did not evaluate. The use of different mouse strains may have the most impact on the difference between the two studies. We have recently reaffirmed that BALB/c mice are much more sensitive to infection than C57BL/6 mice to different strains of *B. pseudomallei* in a murine model of melioidosis [29, 30]. Because of this difference the murine immune response in C57Bl/6 mice to infection is usually more muted than in BALB/c mice which is reflected by a lower recovery of CFU, lower IgG response, suppressed cytokine/chemokine response, and a less cell-mediated host immune response. Still, the heterogeneity in the host response and the development of pyogranulomatous lesions appear to be common in both mouse models. We also reported that IL-1α, IL-1β, KC, and MIG were overall overexpressed besides IFN-γ and TNF-α. Furthermore, we used flow-cytometry to identify the increase in specific inflammatory cells (macrophages, neutrophils, and NK cells) in chronically infected spleens, which was not used in the C57BL/6 study, although neutrophils had been identified earlier from the same group to be the major host cell response required for protection against *B. pseudomallei* in an acute infection model in C57BL/6 mice [62].

Conejero et al. [2015] [63] have further reported on a transcriptome analysis of experimental melioidosis and were able to identify different markers that were associated with acute and chronic infection. There was an upregulation of iNOS in tissue which was reported to be associated with the expression of IFN-γ, and also they found the upregulation of Arginase-1 that was confirmed by immunohistochemistry. Arginase-1 is reported to be an antagonist of iNOS expression. Interestingly, when they compared the transcriptional analysis of tissue and blood between their acute and chronic murine model of melioidosis and that reported for melioidosis in humans, they found common changes in gene expression in their murine model and humans melioidosis patients that included Arginase-1, IL-10, TREM1, and IFN-γ expression.

Although we were able to examine BALB/c mice chronically infected with different human clinical strains of *B. pseudomallei*, there were a number of limitations in our study that should be mentioned. First, we could not control the time we received mice after the initial 21 day LD_50_ aerosol study with different clinical strains of *B. pseudomallei*. Because of the large number of clinical strains that were evaluated, and the large number of mice being examined simultaneously, we could not dictate the time we wanted to examine chronically infected mice after the initial 21 day study. Second, mice that we received were exposed to different amounts of the pathogen. Each strain of *B. pseudomallei* had a different 21 day LD_50_ [although similar in many cases, 29], and different amounts of each strain was used to determine the LD_50_. Third, because of the number of mice we evaluated (~ 100 mice, including controls), we focused our efforts on sera and spleens and did not include lungs. Spleens became infected within 1–2 days after exposure independent of the route of infection so they were a good indicator of the infection process [29–31, 35]. Furthermore, these same studies have reported on the infection occurring in lungs after exposure to a number of different *B. pseudomallei* strains. Fourth, we received a limited number of mice from the exposure group where there might be only a single or a few chronically infected mice because of the heterogeneity in the infection and variation in the virulence of the *B. pseudomallei* strains being evaluated, which may reflect what is seen in the human clinical cases of melioidosis. For this reason we may have a limited amount of information for a particular strain. Lastly, because of the large number of immunological assays and their complexity, we were not able to report on every sample because there may be some inhibition in the assay, or the assay was not complete. However, we examined each surviving mouse that we received separately and evaluated the host immune response to the exposure, and we found that chronically infected mouse had common clinical/pathological features with other chronically infected mice infected with other *B. pseudomallei* strains. Taken together, we have shown that chronically infected BALB/c mice presented a common clinical/immunopathological picture of *B. pseudomallei* infection caused by a number of different strains in the mouse model of chronic melioidosis. Thus, we considered the BALB/c mouse as a good animal model for melioidosis because of the overall host immune response to the pathogen and the clinical presentation after infection.

**Fig. 7:**
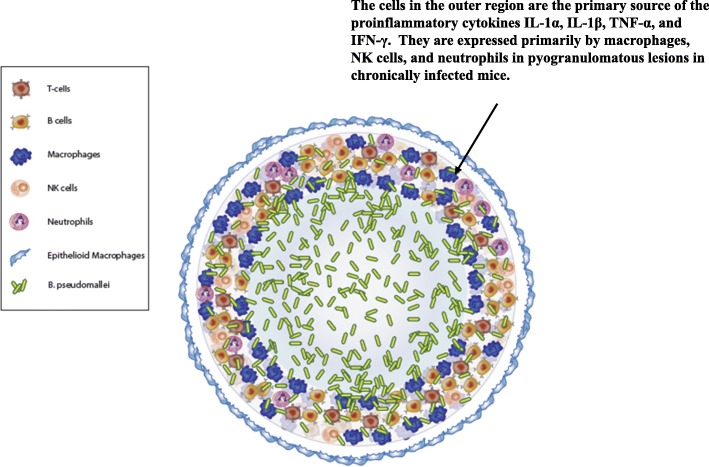
A schematic representation of the *B. pseudomallei* chronic pyogranulomatous lesion. The diagram shows a pyogranulomatous lesion with a central area of necrosis that contains the pathogen which is surrounded by more intact cells. Cells in the latter region consist of a mixture of macrophages, NK cells, neutrophils, and other lymphocytes. The inflammatory cells (macrophages, NK cells, neutrophils) are the main source of TNF-α, IFN-γ, IL-1α, and IL-1β. The expression of the latter two proinflammatory cytokines by inflammatory cells may be in response to signals from cells undergoing necrosis [64], or caspase-1 dependent/independent pyroptosis [65–69]. In many instances we saw a layer of epithelioid macrophages surrounding the pyogranulomatous lesion with some cells that expressed proinflammatory cytokines TNF-α, IFN-γ, IL-1α, and IL-1β

We did not see any marked differences in chronically infected mice (Gp 4) exposed to different strains of *B. pseudomallei*, although we saw some increases in IgG titer and CFU recovered in infected animals that were examined later after exposure, but these results were not consistent. One reason that we may not have observed notable differences in the immune response in mice exposed to the different strains of B. pseudomallei may be because the mice that we examined were chronically infected and not acutely infected. Many differences in the cellular immune response of the host after infection by different *B. pseudomallei* strains occur in the early (acute) phase of infection, especially when examining differences between very virulent B. pseudomallei strains (HBPUB10134a, MSHR5855) and less virulent strains (1106a) [29, 32]. There was a noticeable inverse relationship with the weight of the mouse and size of the chronically infected spleen, however. The normal appearing spleens from exposed BALB/c mice (Gp 2) were not always identical to spleens from naïve mice because we occasionally saw very low humoral and cellular increases in these spleens. In addition, we saw some, albeit low, acquired immune response to the pathogen when we restimulated the cells ex vivo compared to the same cells in media only (see Table 4, Gp 2). The low expression of IFN-γ upon restimulation of splenocytes from these mice with whole cell antigen, suggest that these mice had come in contact with the pathogen. The enlarged spleens (Gp 3) appeared to be either an intermediate state of infection or recovery because in most cases no CFU were recovered from them, but they had an increase in inflammatory cells. Also, sera from mice with enlarged spleens exhibited moderate IgG titers against *B. pseudomallei*, and splenocytes from enlarged spleens produced significant amounts of IFN-γ when restimulated ex vivo, indicating that they had developed both an acquired humoral and cell-mediated immune response to *B. pseudomallei*. We have summarized the observations from our histochemical/immunohistochemical, in situ RNA hybridization, and flow cytometry studies in a model of a pyogranulomatous lesion found in chronically infected mice with a central region of cellular necrosis and debris that is also occupied by *B. pseudomallei* that is similar to the granuloma in tuberculosis (Fig. 7). It shows the presence of a mixture of inflammatory cells (monocytes/ macrophages, NK cells, and neutrophils) and lymphocytes that appear to be more intact in the surrounding outer region of the pyogranulomatous lesion, although a portion may be undergoing necrosis or pyroptosis. The inflammatory cells (macrophages, NK cells, and neutrophils) in this outer regions appeared to be the main source of the proinflammatory/inflammatory cytokines IL-1α, IL-1β, TNF-α, and IFN-γ that we identified in spleens from chronically infected mice. The expression of IL-1α and IL-1β by other inflammatory cells that may be in response to signals from cells undergoing necrosis [64] or caspase-1 dependent/independent pyroptosis [65–69]. The pyogranulomatous lesion may be surrounded by a layer of endothelial macrophages that may be secreting the same inflammatory cytokines. Support for parts of the model of the pyogranulomatous lesion was also from previous reported animal model studies of melioidosis [29–31, 33–35] and tuberculosis [70–72]. There are some similarities between the pyogranulomatous lesions seen in the chronic murine model and lesions reported in humans with chronic melioidosis [30, 73].

Thus, we have further extended the knowledge of the chronic infection state in a murine model of melioidosis. The formation of pyogranulomas in infected organs is common to both murine and human chronic cases of melioidosis, as well as the expression of inflammatory cytokines and acquired immune antibody response after exposure/infection [1, 2], but it is not clear at this time if dysregulation of TNF-α and IFN-γ expression is a common feature that occurs in host cells that are occupied by other intracellular pathogens. This occurrence may be associated with only certain intracellular pathogens, or it may occur only with intracellular pathogens that can mobilize host actin molecules like *B. pseudomallei* which may disrupt the cytosolic milieu. It awaits to be seen if it is a general phenomenon for other intracellular bacterial pathogens. Nevertheless, we believe this observation to be the first report for *B. pseudomallei* (and *B. mallei*, Amemiya et al., unpublished). These common immunological/clinical features that we have identified, which are similar to those seen in cases of human melioidosis, appears in chronically infected mice induced by all *B. pseudomallei* strains that we have examined. These common features can be further used as biomarkers to evaluate different medical countermeasures, such as antibiotic treatments that might show efficacy in the prevention/treatment of chronic melioidosis, or in the discovery of new therapeutic compounds in a mouse model for the treatment of chronic melioidosis in humans. One new class of candidate therapeutics may include checkpoint pathway inhibitors that might have efficacy against the development/treatment of chronic melioidosis.

## Conclusions

We have identified a number of common characteristics in a chronic murine infection model of melioidosis when comparing multiple clinical strains of *B. pseudomallei*. These common features include the demonstration of the presence of the pathogen, the presence of pyogranomatous lesions, the host IgG response to *B. pseudomallei*, the recruitment and enhancement of inflammatory cells in chronically infected mice, the detection of the overexpression of specific proinflammatory cytokines, the ex vivo expression of IFN-γ, TNF-α, and MIP-1α by chronically infected cells, and the detection of intracellular expression of IFN-γ and TNF-α without a protein secretion blocker in inflammatory cells but not in T-cells. Taken together these clinical/immunopathological features extend our knowledge of chronic infection in a BALB/c murine model of melioidosis that can serve as biomarkers to investigate new candidate therapeutic agents for the treatment of chronic melioidosis in humans.

## Methods

### B. Pseudomallei strains

Stock cultures of human clinical isolates of *B. pseudomallei* were collected by the Critical Reagents Program at the US Army Medical Research Institute of Infectious Diseases (USAMRIID) under the guidance of the Defense Threat Reduction Agency and Biomedical Advanced Research and Development Authority (Table 1) [29]. The *Bp* strains were obtained from sources that had the least number of passages after isolation from clinical samples. Single use stock cultures were obtained from the Critical Reagents Program and stored at − 70 °C until used.

### Animal challenges

Groups of female BALB/c mice 6–8 weeks old were obtained from the National Cancer Institute, Frederick, MD. For each aerosol challenge study with a particular strain of *B. pseudomallei*, groups of mice were each placed into five groups with 10 mice per group per housing pan for a total of 50 mice in each study. Mice were housed in clean, autoclaved, filtered top pans, with mouse litter, an enrichment item, and given water and food ad lib., and mouse litter and pans were changed twice a week. Frozen stock cultures of *B. pseudomallei* selected for aerosol challenge were used only once to inoculate 200 ml of 4% glycerol-1% tryptone (BD, Sparks, MD) broth containing 0.5% NaCl (GTB) and incubated overnight for 24 h at 37 °C at 200 rpm. The 24 h culture was diluted to appropriate concentrations in GTB (five concentrations) and used for aerosol challenges by the Center for Aerobiological Science at USAMRIID. Whole-body aerosol exposures of mice were performed as previously described [74] using a 3-jet Collison nebulizer and aerosols monitored with the Automated Bioaerosol Exposure system, after which they are kept in Biosafety Level 3 conditions. Estimation of the amount of aerosol exposure colony forming units (CFU) were determined after each run using the all-glass impinger (AGI), and CFU were determined after an aliquot of each approximately 10-fold dilution of the culture in the AGI was plated in triplicate on sheep blood agar (SBA) plates and incubated at 37^o^ C for 2–3 days.

All animals after challenge were observed twice daily for changes in their condition for the LD_50_ study, and survivors were watched up to 60–70 days post-challenge for changes in appearance. Animals that appeared to be moribund according to an intervention score (which considers overall their appearance, natural behavior, and provoked behavior: Scores of 0–2, normal behavior; 3–7, requires increased monitoring, twice daily; ≥8, moribund) during the study or left over after the studies were humanely euthanized by CO2 inhalation or after anesthesia with a ketamine-acepromazine-xylazine solution (0.1–0.2 ml, given intraperitoneal) and euthanized with a pentobarbital based euthanasia solution given intraperitoneal. Animals that were used in the chronic infection study, were deeply anesthetized with the anesthesia solution given intraperitoneal (0.1–0.2 ml, as stated above), before exsanguination for sera and removal of spleens.

Because of the difficulty in using a set of unexposed, naive mice for every pathogen strain and the different length of time post-infection when animals were examined, we used a group of unexposed 10–12 week old female BALB/c (*n* = 10) mice that served as naïve controls for antibody titers and cytokine/chemokines levels in sera, and spleens for spleen extracts for cytokine/chemokines levels, and splenocytes for cellular composition (flow cytometry), antigen stimulation assays, and intracellular cytokine (TNF-α and IFN-γ) expression. This was done at least twice to obtain normal values for all experimental procedures, but the results of 10 animals were used for statistical analysis when comparing the results of naïve animals with test animals.

All animal use and ethical evaluation had been approved at USAMRIID before starting any proposed animal studies by the Institute IACUC committee, which issued the following statement: Research was conducted under an IACUC approved protocol in compliance with the Animal Welfare Act, PHS Policy, and other federal statutes and regulations relating to animals and experiments involving animals. The facility where this research was conducted is accredited by the Association for Assessment and Accreditation of Laboratory Animal Care, International and adheres to principles stated in the 8th Edition of the Guide for the Care and Use of Laboratory Animals, National Research Council, 2011.

### Antibody determination

Immunoglobulin IgG and subclasses IgG1 and IgG2a levels were determined by ELISA in triplicate and performed at least once as previously described [75]. We did not report the results of samples that did not perform properly, for example we could not obtain an end point to determine the titer. Briefly, irradiated-inactivated *B. pseudomallei* K96243 cells (10 μg/ml in 0.1 M carbonate buffer, pH 9.5), was used to coat a 96-well Immulon 2HB, round-bottom plate (Thermo-Fisher, Pittsburgh, PA) overnight at 4 °C. After washing and blocking the antigen-coated plate, two-fold dilutions of the mouse sera prepared in blocking solution was made in the 96-well plate in triplicate, and plate incubated for 1 h at 37 °C. The plates were then washed and developed with 50 μl of 1/5000 dilution of anti-Ig-horseradish conjugate (Southern Biotechnology Associates, Inc., Birmingham, AL). After the final color development step, the plates were read at 450 nm with a reference filter (570 nm). The results are reported as the reciprocal of the highest dilution giving a mean OD of at least 0.1, which was at least 2-fold over the background ±1 SD, and the final antibody titer was reported as the geometric mean with geometric standard error of the mean (SEM). The results were combined with other mice belonging to the same spleen group, and geometric mean with the standard error of the geometric mean determined. The antibody titer against *B. pseudomallei* K96243 in naïve mice (*n* = 10) was usually below 50, but for statistical purposes it was reported as 50, and it was used to compare with the antibody titer of the mice in the different spleen group for statistical analysis.

### Spleen cell preparation

Splenocytes were prepared from spleens from surviving mice after the 21 day LD50 study was completed as previously described [75]. Briefly, spleens were removed from exsanguinated mice and disaggregated in RPMI 1640 medium (Gibco, Life Technologies, Grand Island, NY) containing 25 mM HEPES and 2 mM glutamine (wash medium) to make the spleen extract. CFU in the freshly prepared extract were determined on sheep blood agar (SBA) plates (BD Diagnostics, Franklin Lake, NJ) with undiluted extract or 10-fold dilutions in sterile water on triplicate SBA plates. Plates were incubated at 37 °C for 2-3 days before counting colonies. The limit of detection was 20 CFU/ml. A portion of the spleen extract was saved for cytokine/chemokine determination after being stored at -70 °C and irradiated to sterilize the extract. An aliquot of the irradiated extract was spread on sheep blood agar plates to check for sterility after 3 days of incubation at 37 °C before use. With the rest of the spleen homogenate, the red cells were lysed with ACK Lysing Buffer (BioWhittaker, Walkersville, MD), and the extract was diluted with wash medium and cells pelleted by centrifugation at 1200 rpm for 10 min. Splenocytes were then washed once and suspended in PBS or complete medium [wash medium containing 10% heat-inactivated fetal calf serum (Life Technologies), 1 mM sodium pyruvate, 0.1 mM non-essential amino acids, 100 U/ml of penicillin, 100 μg/ml streptomycin, and 50 μM 2-mercaptoethanol] and cells counted.

### Cytokine/chemokine expression

Cytokines and chemokines in serum, spleen extracts, and splenocyte culture supernatants were done in duplicate at least once and measured by Luminex MagPix (Life Technologies, Grand Island, NY) as per manufacturer directions using the Mouse Cytokine Magnetic 20-Plex Panel kit. We did not report the results of samples that did not perform properly, for example, if the reading was not within the standard curve. The cytokines/chemokines measured were the following: FGFb, GM-CSF, IFNγ, IL-1α, IL-1β, IL-2, IL-4, IL-5, IL-6, IL-10, IL-12, IL-13, IL-17, IP-10, KC, MCP-1, MIG, MIP-1α, TNFα, and VEGF.

### Splenocyte cellular composition and intracellular cytokine analysis

Approximately 10 × 10^6^ splenocytes prepared from each mouse spleen was suspended in PBS, washed in FACS staining buffer (FSB) (1XPBS, 3% fetal calf serum, Life Technologies), and fixed in FSB containing 4% formaldehyde (Pierce, Rockford, IL) at 4 °C. When Live/Dead Fixable Aqua Dead Cell stain (Molecular Probes, Life Technologies, Eugene, OR) was used, 5 μl of the Aqua Dead Cell stain was added (1 μl per 2 × 10^6^ cells) to the cells in PBS, and cells incubated for 30 min at room temperature protected from light. After incubation, the cells were centrifuged for 10 min at 1200 rpm, and cells were washed once with 1.0 ml of FSB and suspended in 1.0 ml of FSB containing 4% paraformaldehyde and incubated for 1 h at 4 °C. The cells were then washed twice with FSB and suspended in 1.0 ml of FSB. Sterility of the cell suspension was checked by plating 0.1 ml (1/10 volume) onto SBA plates, incubating the plates at 37 °C for 3 days and checked for growth. Cells were then distributed into a microtiter plate (2-5 × 10^5^ cells/well) in duplicate and nonspecific binding was inhibited by the addition of Fc Block (BD Biosciences, San Jose, CA). Cells were labeled with the following specific antibodies (BD Biosciences): CD4 T cells, CD4-PE/CD44-FITC; CD8 T cells, CD8-PE/CD44-FITC; B cells, B220-PE/CD86-FITC; monocytes/macrophages, CD11b-PE/CD44-FITC; NK cells, CD49b-PE/CD44-FITC; and granulocytes, Ly6G-PE/CD44-FITC. Corresponding isotype controls were used and all were incubated for 60 min on ice. Samples were then washed with FSB and incubated with 1X Perm/Wash buffer (BD Biosciences) for 20 min. Intracellular cytokine expression was detected by incubation with anti-IFN-γ-APC or anti-TNF-α-APC for 60 min on ice. All samples were stored in FSB at 4 °C until analysis (and between repeat readings). Cells were identified with a BD FACSCalibur using CellQuestPro software (BDBiosciences). All samples were read at least once. Data was then analyzed with FlowJo Software v10 (FlowJo, Ashland, OR). Gating was established using both fluorochrome isotype controls and single stain controls for each respective assay or against naïve, uninfected splenocytes from BALB/c mice prepared as described above. The results from the exposed mice were placed into groups according to appearance of the spleen (see Fig. 1) and compared to the geometric mean of naïve mice (*n* = 10), unless indicated otherwise, for statistical analysis. We did not report on samples that did not give appropriate readings, such as samples that did not appear to have sufficiently labelled the cell types or have internal labeling perform correctly on infected samples.

### Stimulation of splenocytes

Cytokine/chemokine expression was examined after antigen stimulation of splenocytes prepared from exposed or naive mice by a modification of a procedure previously described [76]. Briefly, 2X10^6^ splenocytes were incubated with either no antigen (media control), 2X10^7^ irradiated, inactivated homologous *B.* p*seudomallei* (challenge strain) bacterial cells, or PMA (100 ng/ml) with ionomycin (500 ng/ml) in duplicate wells of a 48-well, flat bottom, cell culture plate (Costar 3548, Corning, NY) in a final volume of 0.5 ml for 42–46 h at 37 °C + 5% CO_2_. After the incubation period, the cell culture plate was centrifuged at 1200 rpm for 10 min, and the culture supernatant was collected and irradiated to sterilize the sample. Sterility was checked with 0.05 ml of sample plated on a SBA plate, and plate incubated for 3 days at 37 °C. Cytokine/chemokine expression was determined as described above. The combined results from each spleen group is shown in Table 4, and they were compared with the results from the naïve mice group (*n* = 2) for statistical analysis. Not all samples were analyzed in this manner because the assay was not performed until the study was underway.

### Immunohistochemistry and in situ hybridization

Spleens from mice were placed into 10% neutral buffered formalin for at least 21 days before they were taken out of biosafety level 3 conditions. The spleens were embedded in paraffin and sectioned for staining with hematoxylin and eosin (H&E). For immunohistochemical analysis, a rabbit polyclonal antibody raised against an extract of *B. pseudomallei* K96243 (a kind gift from Dr. Robert Ulrich) was used to identify the *B. pseudomallei* exopolysaccharide antigen in formalin-fixed, paraffin-embedded tissue [30, 35].

To examine the expression of cytokines (IL-1α, IL-1β, TNF-α, and IFN-γ) in formalin-fix, paraffin-embedded tissue, a RNAscope technology (Advance Cell Diagnostics, Inc., Hayward, CA) was used that targets the RNA of the cytokine of interest by in situ hybridization (ISH) [36]. In brief, tissue sections were fixed onto slides and pretreated to unmask target RNA and cells permeabilized with RNAscope Pretreatment solutions. Special designed proprietary paired Z-probes that hybridized to adjacent RNA sequences ensured the specificity of the amplified signal. It was unlikely that adjacent sequences that are targeted by two separate probes would be found on another RNA target. There were up to 20 paired Z-probes for each targeted (cytokine) RNA. After the initial hybridization, pre-amplifiers hybridized to paired Z-probes which were followed by amplifiers that bound to multiple binding sites on each preamplifier. Labeled probes that contain a chromogenic enzyme bound to multiple sites on each amplifier**.** After incubation with a red substrate-chromogen solution for 10 min at room temperature, sections were then counter stained with hematoxylin, air dried, and mounted. A bright field microscope was used to examine the stained specimens. See the individual Figure Legends for the number of test samples used that was compared to the samples from a naïve mouse.

### Statistical analyses

Log transformation was applied to cytokine/chemokine concentrations (in sera, cell extracts, culture supernatants, and intracellular), antibody titers, and cell distribution prior to analysis, with comparisons between selected group means made by Welch’s t-test. Results were summarized as geometric mean and geometric standard error. No multiple comparison adjustment has been applied to the reported *p*-values. Where detection limits were encountered in cytokine/chemokine data, the limit of detection was imputed. Analysis was implemented in SAS version 9.4, SAS Institute Inc., Cary, NC, USA. Results were considered significant when *P <* 0.05.

## Supplementary information

**Additional file 1 Figure S1**. Schematic of analysis of chronically infected BALB/c mice. Processing of mouse samples for analysis of chronic infection.

**Additional file 2 Table S1**. Cytokines/chemokines in sera and spleen extracts in different groups of mice. A. Serum levels. B. Spleen extract levels.

**Additional file 3 Figure S2**. Intracellular expression of TNF-α and IFN-γ in infected spleen cells from *B. pseudomallei* exposed mice. The data are representative of 49 spleens from *B. pseudomallei* (includes 8 different strains) exposed mice and 10 naïve mice that were determined at least once. The aerosol 21 day LD_50_ studies of some *B. pseudomallei* strains were evaluated more than once. Examples of the intracellular expression of TNF-α in different cell types is shown in Figure S2A**.** Cytometric analysis of spleens from a naïve mouse (panel 1), two infected spleens from mice exposed to 7007 CFU of *B. pseudomallei* 1106a 50 days PI (panel 2), two spleens from mice exposed to ~ 1 CFU of *B. pseudomallei* MSHR5855 26 days PI, one normal appearing and one infected spleen (panel 3), and two spleens from mice exposed to 2 CFU of *B. pseudomallei* 1026b 30 days PI, one swollen and one infected (panel 4). The intracellular expression of IFN-γ in the same spleen cells is shown in Figure S2B. The intracellular expression of TNF-α and IFN-γ was examined in CD4+/CD44+ and CD8+/CD44+ T Cells, B cells (B220+/CD86+), monocyte/macrophages (CD11b+/CD44+), NK cells (CD49b+/CD44+), and granulocytes (Ly6G+/CD44+). The gating for the percentage of cells with intracellular cytokine expression was established with isotype or single stained controls or against naïve mice. Note the greater changes in the % monocyte/macrophages, % NK cells, and % granulocytes population in the cells from infected spleens.

## Data Availability

All data used and analyzed during this study are included within this article and supporting files.

## Abbreviations

AGI: All glass impinger
AIDs: Acquired immunodeficiency syndrome
ATCC: American Type Culture Collection
BFA: Brefeldin A
Bp: Burkholderia pseudomallei
CFU: Colony forming units
CTLA-4: Cytotoxic T lymphocyte associated protein 4
ELISA: Enzyme-linked immunosorbant assay
ER: Endoplasmic reticulum
FACS: Flurorescent activated cell sorter
FSB: FACS staining buffer
GM-CSF: Granuylocyte-macrophage colony-stimulating factor
Gp: Group
GTB: Glycerol tryptone broth
H&E: Haemotoxylin and eosin
HIV: Human Immunodeficiency virus
IACUC: Institutional Animal Care and Use Committee
IFN-γ: Interferon –gamma
IgG: Immunoglobulin G
IHC: Immunohistochemistry
IL: Interleukin
IP-10: Interferon-gamma-induced protein 10
KC: Keratinocyte-derived chemokine
LD50: Median lethal dose
MCP-1: Monocyte chemoattractant protein-1
MHC: Major histocompatibility complex
MIG: Monokine induced by gamma interferon
MIP-1α: Macrophage inflammatory protein-1 alpha
MyD88: Myeloid differentiation primary response 88 protein
NK: Natural Killer
PAMPs: Pathogen associated molecular patterns
PBS: Phosphate buffered saline
PD-1: Program cell death 1
PD-L1: Program cell death ligand-1
PI: Post-infection
SBA: Sheep blood agar
SCV: Small colony variant
SEM: Standard error of the mean
Th-1: T helper-1
Th-2: T helper-2
TIM3: T cell immunoglobulin and mucin domain containing-3
TLR: Toll-like receptor
TNF-α: Tumor necrosis factor-alpha
USAMRIID: US Army Medical Research Institute of Infectious Diseases
VEGF: Vascular endothelial growth factor
