# Supplementary material for: Dysregulation of TNF-α and IFN-γ expression is a common host immune response in a chronically infected mouse model of melioidosis when comparing multiple human strains of Burkholderia pseudomallei
Source: BMC Immunol. 2020 Feb 3;21:5. doi: 10.1186/s12865-020-0333-9 (PMC6998218; doi:10.1186/s12865-020-0333-9)
Supplement: Supplementary file 1 — Additional file 1 Figure S1. Schematic of analysis of chronically infected BALB/c mice. Processing of mouse samples for analysis of chronic infection. [file 12865_2020_333_MOESM1_ESM.pptx]

## Slide 1
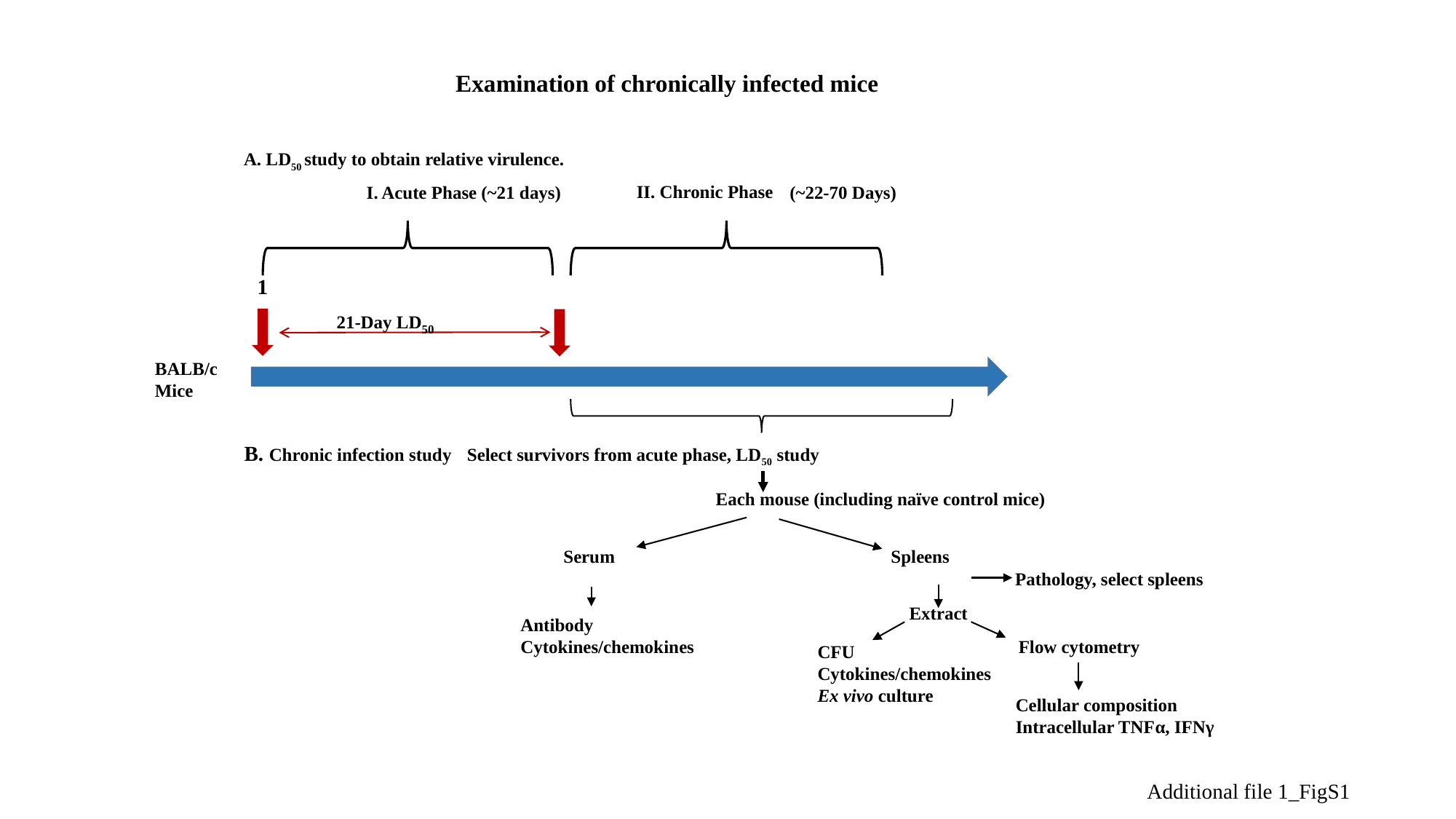

Examination of chronically infected mice
A. LD50 study to obtain relative virulence.
II. Chronic Phase
I. Acute Phase (~21 days)
(~22-70 Days)
1
21-Day LD50
BALB/c
Mice
B. Chronic infection study	 Select survivors from acute phase, LD50 study
Each mouse (including naïve control mice)
Serum			Spleens
Pathology, select spleens
Extract
Antibody
Cytokines/chemokines
Flow cytometry
CFU
Cytokines/chemokines
Ex vivo culture
Cellular composition
Intracellular TNFα, IFNγ
Additional file 1_FigS1
