# Supplementary figures and images for: Dysregulation of TNF-α and IFN-γ expression is a common host immune response in a chronically infected mouse model of melioidosis when comparing multiple human strains of Burkholderia pseudomallei
Source: BMC Immunol. 2020 Feb 3;21:5. doi: 10.1186/s12865-020-0333-9 (PMC6998218; doi:10.1186/s12865-020-0333-9)

1. **TNF-α.**


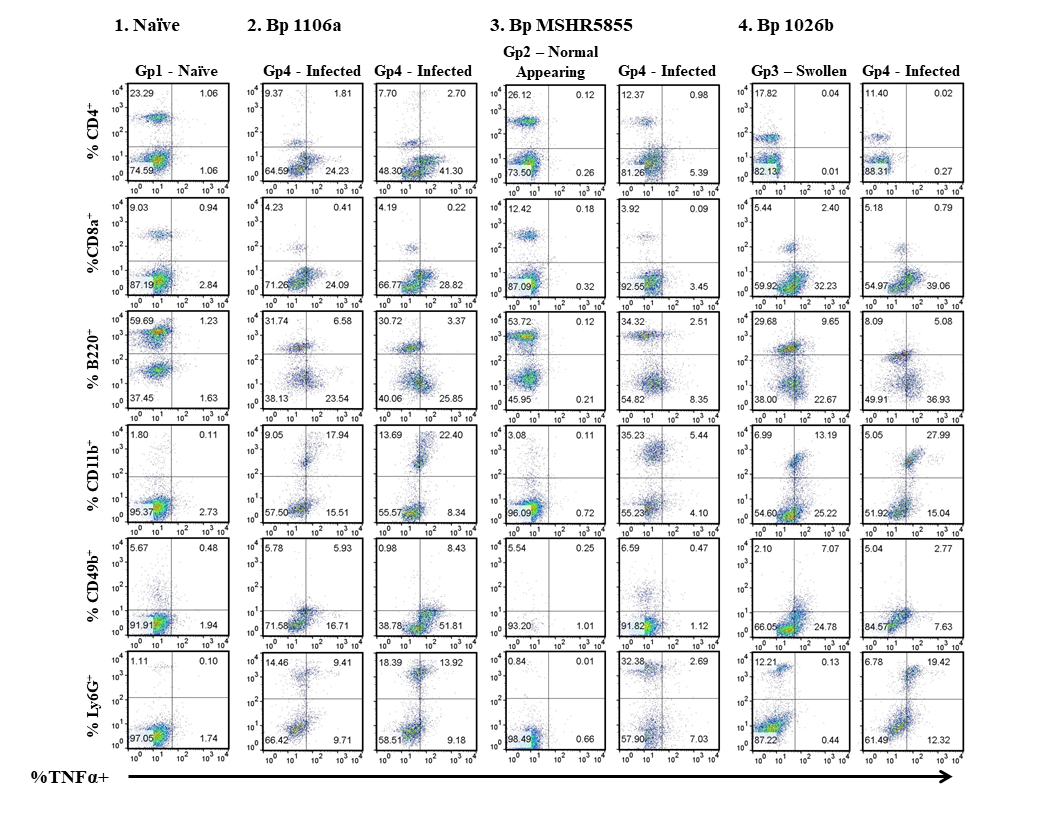


1. **IFN-γ**


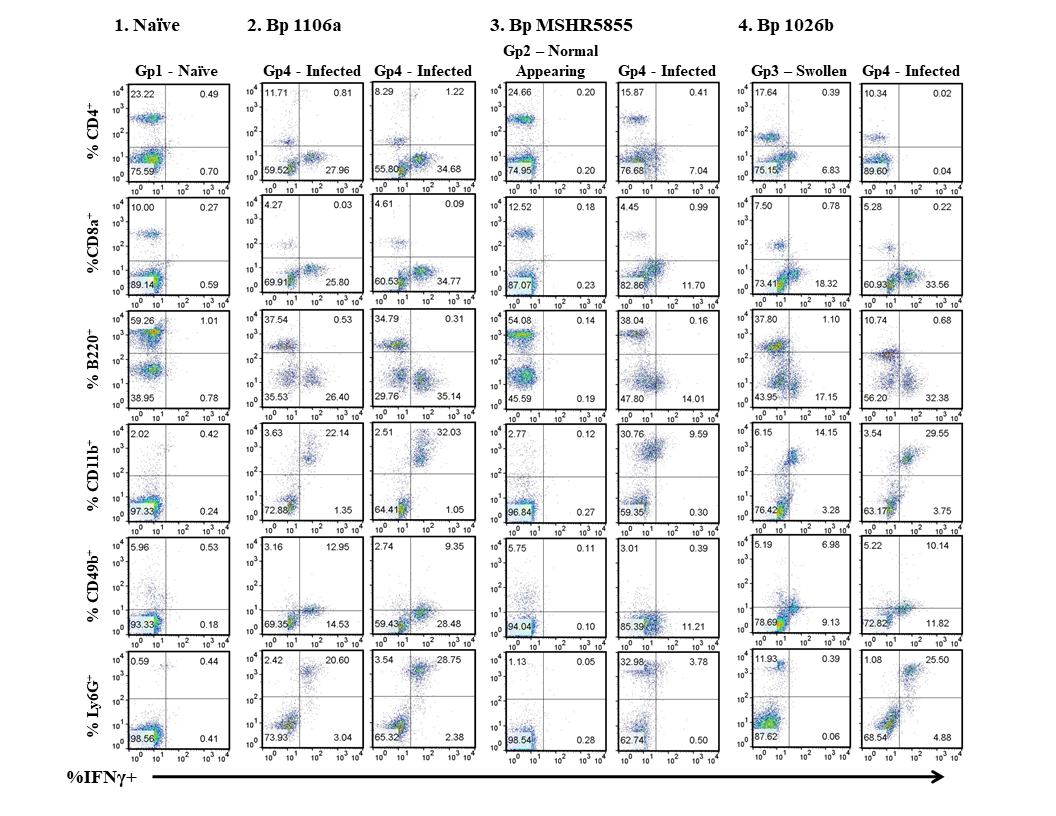


**Fig. S2.**

Supplement: Supplementary file 3 — Additional file 3 Figure S2. Intracellular expression of TNF-α and IFN-γ in infected spleen cells from B. pseudomallei exposed mice. The data are representative of 49 spleens from B. pseudomallei (includes 8 different strains) exposed mice and 10 naïve mice that were determined at least once. The aerosol 21 day LD50 studies of some B. pseudomallei strains were evaluated more than once. Examples of the intracellular expression of TNF-α in different cell types is shown in Figure S2A. Cytometric analysis of spleens from a naïve mouse (panel 1), two infected spleens from mice exposed to 7007 CFU of B. pseudomallei 1106a 50 days PI (panel 2), two spleens from mice exposed to ~ 1 CFU of B. pseudomallei MSHR5855 26 days PI, one normal appearing and one infected spleen (panel 3), and two spleens from mice exposed to 2 CFU of B. pseudomallei 1026b 30 days PI, one swollen and one infected (panel 4). The intracellular expression of IFN-γ in the same spleen cells is shown in Figure S2B. The intracellular expression of TNF-α and IFN-γ was examined in CD4+/CD44+ and CD8+/CD44+ T Cells, B cells (B220+/CD86+), monocyte/macrophages (CD11b+/CD44+), NK cells (CD49b+/CD44+), and granulocytes (Ly6G+/CD44+). The gating for the percentage of cells with intracellular cytokine expression was established with isotype or single stained controls or against naïve mice. Note the greater changes in the % monocyte/macrophages, % NK cells, and % granulocytes population in the cells from infected spleens. [file 12865_2020_333_MOESM3_ESM.docx]
